# Maternal Phthalate Exposure and Allergic Diseases in Children: A Meta-Analysis and Network Toxicology

**DOI:** 10.3390/ijms26136103

**Published:** 2025-06-25

**Authors:** Yi Xiang, Yanming Lv, Wenhao Fu, Jie Wen, Baixiang Li, Xueting Li

**Affiliations:** Department of Hygienic Toxicology, School of Public Health, Harbin Medical University, 157 Baojian Road, Harbin 150081, China; xy1790942103@163.com (Y.X.); lvyanming2023@163.com (Y.L.); 18219773672@163.com (W.F.);

**Keywords:** meta-analysis, phthalates, allergic diseases, prenatal exposure, postnatal exposure

## Abstract

Several studies suggest a relationship between phthalates (PAEs) and allergic diseases in children. Therefore, we speculated that PAE exposure may be an important environmental factor causing allergic diseases. The present study employed meta-analysis and network toxicology to analyze the interactions and assess potential pathogenic pathways between prenatal and postnatal PAE exposure and childhood allergic diseases. This study found that prenatal PAEs exposure was positively associated with childhood wheezing and eczema (OR = 1.03, 1.05), and postnatal PAEs exposure was positively associated with childhood wheezing, eczema, and rhinitis (OR = 1.10, 1.05, 1.06). PAE exposure from dust may elicit distinct effects compared to direct exposure to PAEs. Furthermore, a large number of overlapping genes between disease targets and PAEs were identified. Enrichment analysis highlighted the association of PAE-targeted genes with biological pathways integral to allergic diseases. Molecular docking results indicated a strong link between the PAEs and the core proteins, such as SRC, AKT1, and HSP90AA1. These proteins are critically involved in the regulation of immune–inflammatory processes underlying allergic diseases. This discovery not only enhances our understanding of the relationship between environmental pollutants and child health but also provides a robust reference for experimental studies on the induction of childhood diseases by early-life exposure to environmental pollutants.

## 1. Introduction

Phthalates (PAEs), as an environmental endocrine disruptor, are widely utilized as plasticizers in plastic tubing, materials for food packaging, and various medical devices [1]. Apart from being employed as plasticizers, PAEs are also utilized in various personal care products, including perfumes, soaps, and other items for fragrance purposes. It does not chemically combine with other substances in these products, so it can easily be released into the peripheral environment, causing environmental pollution and health hazards. It is widely exposed to humans through ingestion, inhalation, and dermal contact, thus ultimately causing a wide range of negative effects on human health, such as reproductive toxicity, cardiovascular toxicity, and neurotoxicity [2]. There are 13 PAEs included in this research; they are as follows: Mono(carboxyisooctyl) phthalate (MCOP), Mono(2-ethyl-5-hydroxyhexyl) phthalate (MEHHP), Mono(2-ethyl-5-oxohexyl) phthalate (MEOHP), Mono(2-ethyl-5-carboxypentyl) phthalate (MECPP), Monoethyl phthalate (MEP), Mono-isobutyl phthalate (MiBP), Mono(2-ethylhexyl) phthalate (MEHP), Mono-n-butyl phthalate (MnBP), Mono(3-carboxypropyl) phthalate (MCPP), Monobutyl phthalate (MBP), mono-Benzyl phthalate (MBzP), DiEthyl Hexyl Phthalate (DEHP), and Mono (7-carboxy-2, 7-dimethylheptyl) Phthalate (MCNP).

Evidence links PAE exposure to increased risk of allergic diseases. Allergic diseases are a category of systemic diseases generated by dysregulation of innate and adaptive immune responses, including asthma, rhinitis, and eczema [3,4]. It is estimated that 12% of children suffer from asthma, 9% of children suffer from allergic rhinitis, and 22% suffer from eczema worldwide, which imposes a serious health and economic burden [5]. Asthma is characterized by airway inflammation resulting in respiratory dysfunction [6]. Eczema is also called atopic dermatitis. It is a common skin disease that often presents with dry skin, itchiness with pain, sleep disturbances, and depression [7]. The typical symptoms of allergic rhinitis consist of sneezing, itching, runny nose, and nasal congestion [8]. The recent test of asthma symptoms conducted by the Global Asthma Network (GAN) using the ISAAC protocol found that 6.9% of adolescents worldwide have severe asthma symptoms [9]. It is estimated that nearly 500 million people worldwide have allergic rhinitis [10]. Wheezing is a common problem in children’s respiratory systems. The causes are complex, and in severe cases, it will affect motor ability and daily learning. Persistent wheezing may progress to asthma. However, not all childhood wheezing progresses to asthma [11]. The pathogenesis of various types of allergic diseases remains largely unknown. Therefore, the identification of risk factors for allergic diseases can be of great help in disease prevention. In addition to genetic susceptibility, environmental factors are associated with the development of allergic diseases [12].

Several epidemiologic studies have found an increased risk of allergic disease in children postnatally exposed to PAEs. A population study in Shanghai found a positive association between urinary MnBP concentrations and the development of wheezing, rhinitis, and eczema in children [3]. In addition, the EDEN Mother–Child Cohort Study in 2018 from France demonstrated that the metabolites of PAEs were significantly associated with eczema risk [13]. The developmental origins of health and disease (DOHaD) hypothesis suggests that children are exposed to environmental factors in utero and during early development that predispose them to developmental abnormalities and increase susceptibility to disease [14]. Berger et al. in 2020 also found that prenatal exposure to PAEs increased the risk of childhood asthma [15].

Research on PAEs has, thus far, mostly concentrated on their correlation with poor health outcomes in adults. There is virtually little research on the connection between PAEs and adverse health outcomes in children, particularly in relation to allergic diseases. Therefore, we conducted this comprehensive study, which included an analysis of 54 articles, to explore the connection between maternal exposure to PAEs (both prenatally and postnatally) and the incidence of allergic diseases in children. To comprehensively examine the deleterious effects of PAEs on allergic diseases, we employed network toxicology and molecular docking techniques. Our work provides a scientific basis for developing more precise monitoring and intervention strategies for hazardous environmental pollutants. Additionally, it offers crucial clues for the prevention of asthma and other diseases, as well as for the search and development of associated drugs.

## 2. Results

### 2.1. Study Characteristics of Included Literature for Meta-Analysis

**Figure 1:**
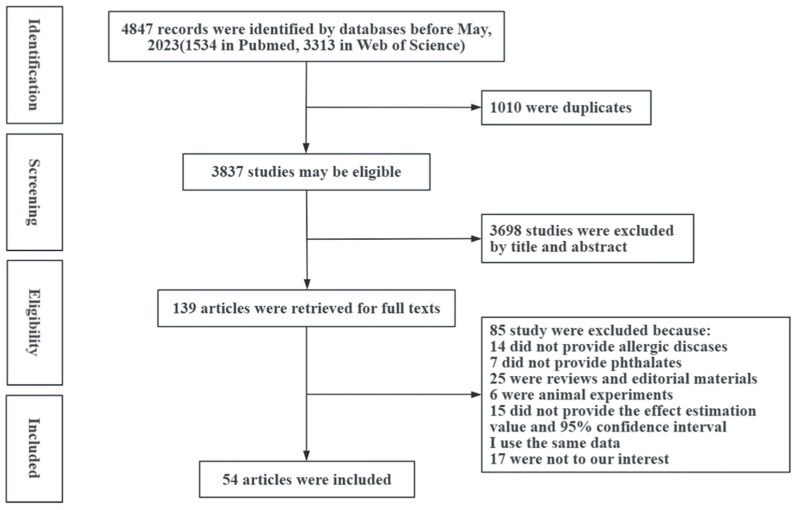
Flowchart of study search and selection.

Out of the 4847 studies found in the electronic database, 54 were selected for further analysis following screening. Figure 1 shows the general flow of the study screening. Among these 54 included studies [3,10,13,16,17,18,19,20,21,22,23,24,25,26,27,28,29,30,31,32,33,34,35,36,37,38,39,40,41,42,43,44,45,46,47,48,49,50,51,52,53,54,55,56,57,58,59,60,61,62,63,64,65,66], the impacts of PAEs on wheezing (24/54 studies), asthma (30/54 studies), eczema (29/54 studies), and rhinitis (19/54 studies) were assessed (Table S1). The case–control, cross-sectional, and cohort studies, respectively, accounted for 14.8%, 25.9%, and 59.3%.

### 2.2. Targets of PAEs and Allergic Diseases

A total of 2278 asthma targets, 958 rhinitis targets, 771 eczema targets, and 771 wheezing targets were collected from GeneCards and OMIM databases. Additionally, 421 DEHP targets, 393 MBzP targets, 393 MCOP targets, 368 MEHHP targets, 396 MEOHP targets, and 394 MECPP targets were identified from three databases: Pharmmapper, SwissTargetPrediction, and TargetNet.

### 2.3. Effects of Prenatal and Postnatal Phthalate Exposure on Wheezing in Children

Ten studies provided the link between prenatal PAE exposure and childhood wheezing. We discovered that wheezing was more common in children whose mothers had been exposed to PAEs during pregnancy (OR 1.03, 95% confidence interval 1.00–1.06) (Figure S1). However, the analysis of specificity for the 13 PAE subtypes did not reveal statistically significant correlations (*p* < 0.05), and subgroup analyses suggested that the heterogeneity of MEHHP, MEOHP, and MECPP exposures was mainly related to (1) late mid- and late-pregnancy exposures (I^2^ = 72.2–80.4%); (2) North American populations (I^2^ = 87.3–92.0%); and (3) multiple pregnancies (I^2^ = 72.2–80.4%) (Table S3).

**Figure 2:**
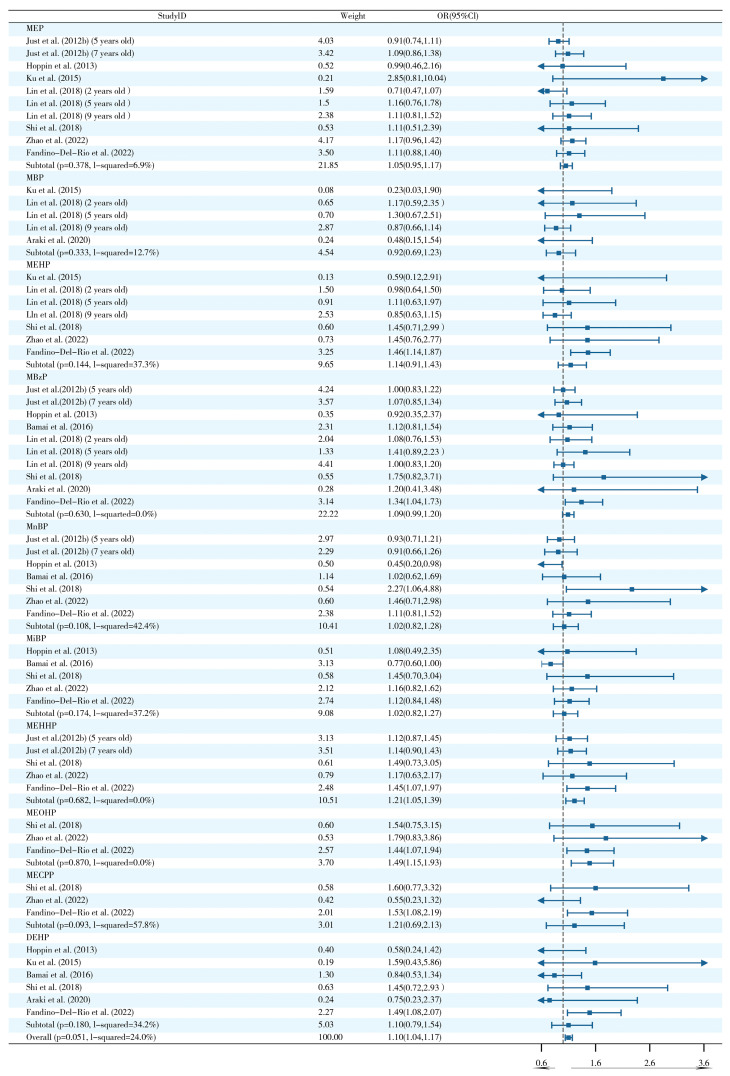
Forest plots of postnatal PAE exposure and wheezing [3,17,21,30,34,38,39,45,48,65].

Evidence of a correlation between postnatal exposure to PAEs and wheezing was found in nine studies. A pooled analysis based on nine studies demonstrated that postnatal PAE exposure significantly increased the risk of wheezing (OR = 1.10, 95% CI: 1.04–1.17) (Figure 2). Notably, MEHHP (OR = 1.21, 95% CI: 1.05–1.39) and MEOHP (OR = 1.49, 95% CI: 1.15–1.93) subtypes showed specific dose–effect relationships. Subgroup analysis revealed significant heterogeneity in MnBP exposure in the North American population (I^2^ = 76.7%, *p* = 0.038) (Table S4), suggesting that geographic factors may influence the biological effects of this subtype. 

Five studies addressing indoor dust exposure pathways showed a trend towards a non-significant positive association between PAE exposure and wheezing risk (OR = 1.07, 95% CI: 0.97–1.18) (Figure S2). Although none of the analyses for the five main dust sources of PAEs, including MEP, MnBP, and MiBP, reached the threshold of statistical significance (*p* < 0.05), subgroup analyses suggested that different demographic characteristics may influence the heterogeneity of exposure effects (Table S5).

**Figure 3:**
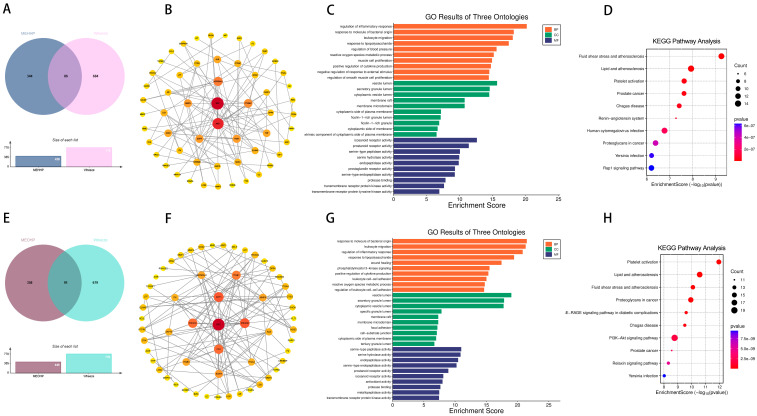
Venn diagram of potential targets of toxicity of MEHHP to wheezing (**A**); PPI of potential targets of toxicity of MEHHP to wheezing (**B**); GO (**C**) and KEGG (**D**) enrichment analyses of potential targets of toxicity of MEHHP to wheezing. Venn diagram of potential targets of toxicity of MEOHP to wheezing (**E**); PPI of potential targets of toxicity of MEOHP to wheezing (**F**); GO (**G**) and KEGG (**H**) enrichment analyses of potential targets of MEOHP to wheezing.

To further determine the relationship between wheezing and the two previously mentioned PAEs (MEHHP and MEOHP). We identified 86 targets of MEHHP and wheezing and 91 overlapping genes between MEOHP and wheezing, respectively (Figure 3A,E). Notably, SRC, AKT1, and HSP90AA1 and SRC, AKT1, and PIK3CB were identified as the top three core targets of the two PAEs, respectively, based on the extent value of the targets in the protein interaction network (Figure 3B,F). Shared terms between the two datasets highlighted core pathways associated with immune and inflammatory regulation, including “regulation of inflammatory response” (BP), “leukocyte migration” (BP), and “reactive oxygen species metabolic process” (BP), alongside vesicle trafficking compartments (CC) and protease-related functions (MF). Distinctively, MEHHP prioritized terms linked to pathogen response and tissue repair (BP), whereas MEOHP emphasized vascular/muscular adaptation (BP) and growth signaling (MF). The results of KEGG enrichment analysis further provided support for the GO results (Figure 3C,D,G,H). We assessed the binding between MEHHP and MEOHP and the corresponding core targets by molecular docking, and the Vina scores in the results were all less than −5, suggesting that the binding was favorable (Table 1).

### 2.4. Effects of Prenatal and Postnatal Phthalate Exposure on Asthma in Childhood

Fourteen studies provided the association of prenatal PAEs exposure and childhood asthma. The pooled OR value of prenatal PAE exposure for childhood asthma risk was 1.02 (OR = 1.02, 95% CI: 0.99–1.06) (Figure S3). However, none of the subtype analyses for the 13 PAE metabolites achieved statistical significance (*p* < 0.05). Subgroup analyses revealed that the heterogeneity of MBP exposure originated from early pregnancy (I^2^ = 62.3%, *p* = 0.047) and the Oceanian population (I^2^ = 77.6%, *p* = 0.034). By contrast, the heterogeneity of MBzP and MEHP was mainly associated with early pregnancy exposure and Asian geographic factors (I^2^ = 60.8–62.1%, *p* < 0.05) (Table S6).

Eleven research studies that looked into the relationship between postnatal exposure to PAEs and asthma were found. The pooled OR value of postnatal PAE exposure for asthma risk was 1.04 (95% CI: 0.95–1.14) (Figure S4). Although specific analyses of 13 PAE subtypes did not reveal significant associations, subgroup analyses suggested significant heterogeneity in MnBP exposure in European populations (I^2^ = 63.0%, *p* = 0.044) (Table S7).

**Figure 4:**
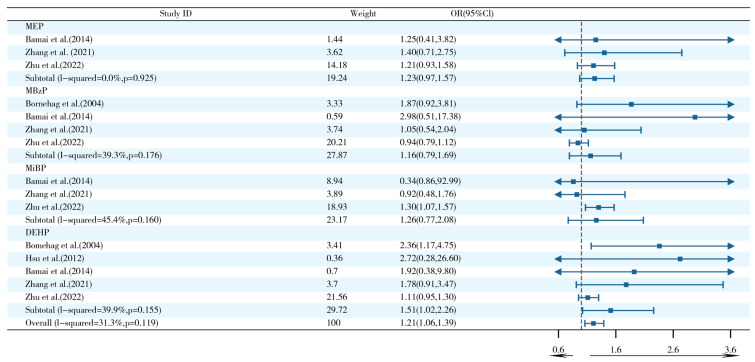
Forest plots of postnatal exposure to PAEs from indoor dust and asthma [1,20,25,35,63].

The five studies focusing on exposure pathways found that exposure to PAEs from indoor dust sources was significantly associated with an increased risk of asthma (OR = 1.21, 95% CI: 1.06–1.39) (Figure 4). It is noteworthy that the DEHP subtype exhibited a discernible dose–effect relationship (OR = 1.51, 95% CI: 1.02–2.26), while the remaining PAEs did not demonstrate statistically significant associations. Subgroup analyses were further conducted in this study but did not explain the heterogeneity of the results (Table S8).

**Figure 5:**
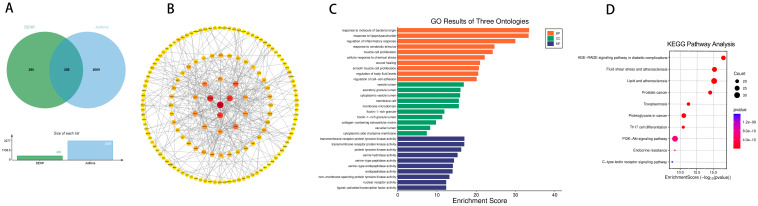
Venn diagram of potential targets of toxicity of DEHP to asthma (**A**); PPI of potential targets of toxicity of DEHP to asthma (**B**); GO (**C**) and KEGG (**D**) enrichment analyses of potential targets of toxicity of DEHP to asthma.

To further elucidate the association between DEHP and asthma, a significant overlap of 208 genes between DEHP and asthma was identified (Figure 5A). The PPI network of overlapping genes identified SRC, AKT1, and HSP90AA1 as the three most critical core targets (Figure 5B). GO enrichment analysis was combined with the results of KEGG pathway enrichment analysis, and these findings suggest that these shared genes play key roles in immune responses, extracellular structures and activities, and signaling pathways associated with cardiovascular disease and cancer (Figure 5C,D). To investigate the interaction of DEHP with core target genes (SRC, AKT1, and HSP90AA1), molecular docking analyses were performed. Notably, the corresponding core target proteins exhibited strong binding affinity for DEHP, with respective Vina scores below −5.0. These results highlight significant binding interactions between DEHP and these core targets, suggesting that they may be involved in the molecular mechanism of DEHP-induced asthma (Table 1).

### 2.5. Effects of Prenatal and Postnatal Phthalate Exposure on Eczema in Children

A synthesis of 14 studies revealed a marginal yet significant association between PAE exposure and elevated infantile eczema risk (pooled OR = 1.05, 95% CI: 1.01–1.08) (Figure S5). Subtype analyses identified MBzP as a clinically relevant metabolite, demonstrating a 17% increased eczema risk per exposure unit (OR = 1.17, 95% CI: 1.03–1.32). Amongst the 13 assessed PAE metabolites (MEP, MEHP, DEHP, etc.), no additional significant correlations were observed. Subgroup analyses revealed substantial heterogeneity in the effects of prenatal MEHHP (I^2^ = 86.6%, *p* = 0.06) and MEOHP (I^2^ = 81.5%, *p* = 0.02) exposure (Table S9).

**Figure 6:**
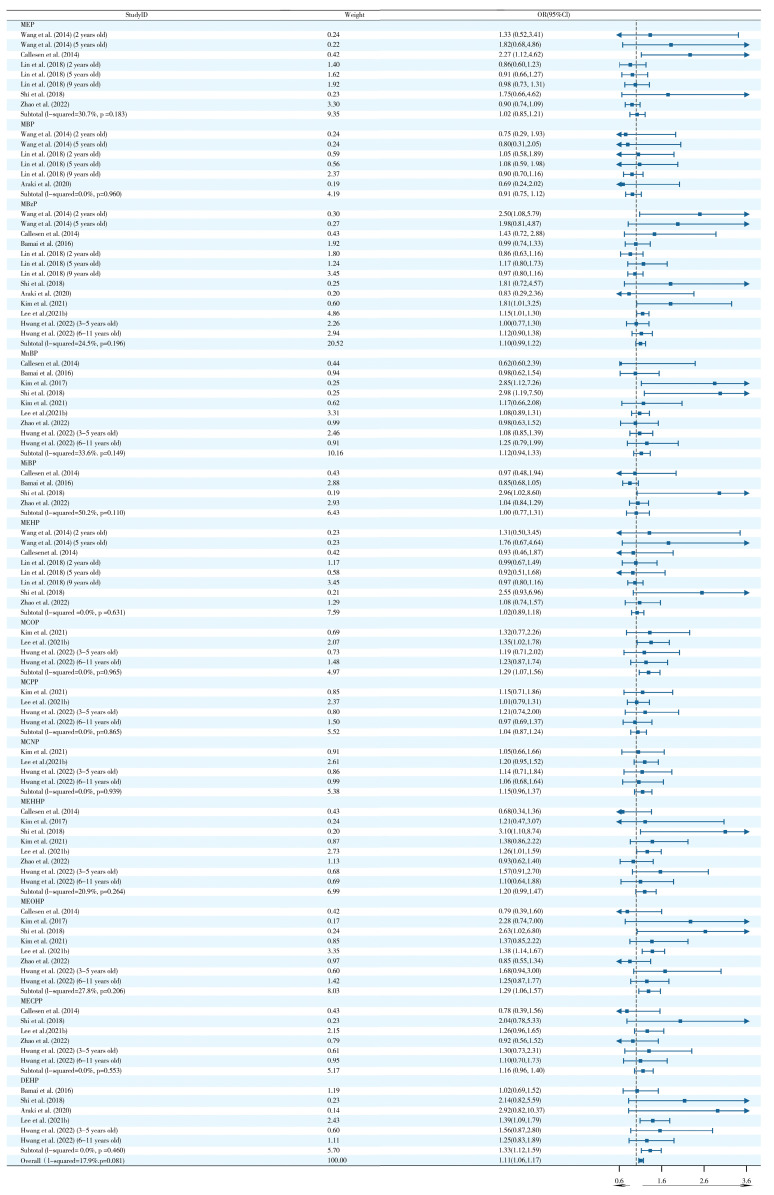
Forest plots of postnatal PAE exposure and eczema [3,17,21,27,36,42,43,47,48,59,65].

11 studies indicated a significant dose-dependent relationship between postnatal PAE exposure and eczema risk (OR = 1.11, 95% CI: 1.06–1.17) (Figure 6). Further analyses identified MEOHP (OR = 1.29, 95% CI: 1.06–1.57) and DEHP (OR = 1.33, 95% CI: 1.12–1.59) as key risk metabolites assessed PAEs. Subgroup analyses revealed significant heterogeneity for MBzP exposure in families with <3 children (I^2^ = 81.8%, *p* = 0.019) and MiBP exposure in Asian populations (I^2^ = 66.8%, *p* = 0.049) (Table S10).

A series of six studies were conducted to evaluate the association between indoor dust PAE exposure pathways and the development of eczema (Figure S6). The results of these studies indicated that there was no significant association between PAEs and eczema (OR = 1.01, 95% CI: 0.93–1.09). Notably, postnatal exposure to a specific class of PAEs from indoor dust was not significantly associated with eczema. Subgroup analyses revealed significant heterogeneity in MBzP effects within Asian cohorts (I^2^ = 74.0%, *p* = 0.009) and MiBP associations in cross-sectional designs (I^2^ = 84.8%, *p* = 0.01) (Table S11).

**Figure 7:**
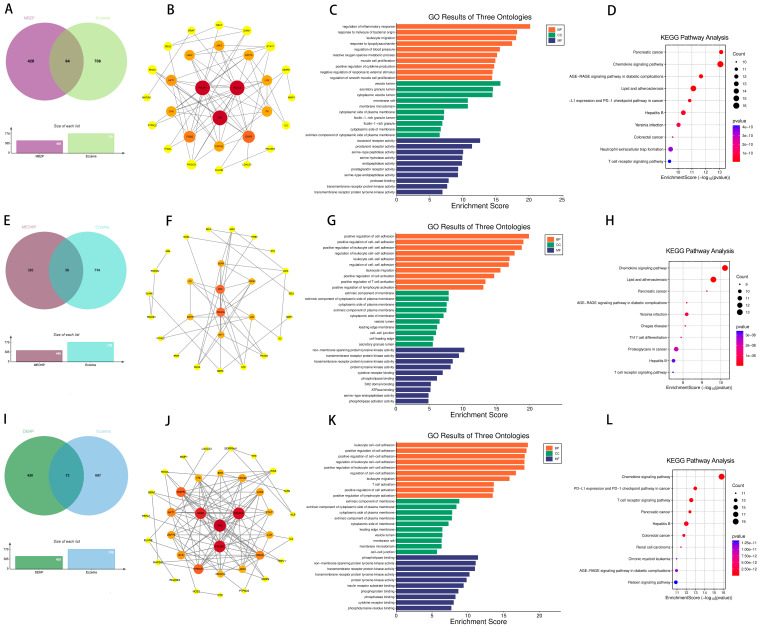
Venn diagram of potential targets of toxicity of MBzP to eczema (**A**); PPI of potential targets of toxicity of MBzP to eczema (**B**); GO (**C**) and KEGG (**D**) enrichment analyses of potential targets of toxicity of MBzP to eczema. Venn diagram of potential targets of toxicity of MEOHP to eczema (**E**); PPI of potential targets of toxicity of MEOHP to eczema (**F**); GO (**G**) and KEGG (**H**) enrichment analyses of potential targets of toxicity of MEOHP to eczema. Venn diagram of potential targets of toxicity of DEHP to eczema (**I**); PPI of potential targets of toxicity of DEHP to eczema (**J**); GO (**K**) and KEGG (**L**) enrichment analyses of potential targets of toxicity of DEHP to eczema.

Furthermore, further analyses revealed 73, 64, and 56 overlapping genes between DEHP, MBZP, MEOHP, and eczema, respectively (Figure 7). Subsequently, this study constructed a PPI network of these overlapping genes, and it is noteworthy that the three different species of PAEs have the same core targets, i.e., SRC, PIK3R1, and PIK3CA. The intersection of genes of MBzP, MEOHP, and DEHP with eczema pathogenesis highlighted a convergence on immune–inflammatory pathways, including “leukocyte cell–cell adhesion” (BP), “positive regulation of leukocyte adhesion to vascular endothelial cell” (BP), and “T cell activation” (BP), underscoring dysregulated immune cell trafficking and activation. Shared cellular components such as “extrinsic component of cytoplasmic side of plasma membrane” (CC) and “vesicle lumen” (CC) implicated membrane-associated signaling and secretory processes, while molecular functions like “transmembrane receptor protein tyrosine kinase activity” (MF) and “cytokine receptor binding” (MF) suggested kinase-driven inflammatory cascades. Notably, DEHP-specific terms (e.g., “phosphatidylinositol 3–kinase signaling”) aligned with cell survival modulation, whereas MBzP and MEOHP uniquely enriched “reactive oxygen species metabolic process” (BP) and “phospholipase binding” (MF). KEGG pathway enrichment results revealed that the intersection genes of DEHP, MEOHP, and MEHHP with eczema pathogenesis revealed significant enrichment in pathways central to immune dysregulation and inflammatory cascades. Shared pathways, including the “chemokine signaling pathway” and “T cell receptor signaling pathway”, underscored aberrant leukocyte recruitment and T-cell-mediated immune activation, aligning with eczema’s inflammatory phenotype. The molecular docking results were used to suggest the binding of the three PAEs mentioned above to the core target, and the binding energies were all less than −5, so we can consider the binding to be very robust (Table 1).

### 2.6. Effects of Prenatal and Postnatal Phthalate Exposure on Rhinitis in Children

A meta-analysis of four studies revealed that the association between prenatal PAE exposure and childhood rhinitis was not significant (OR = 1.05, 95% CI: 0.97–1.14) (Figure S7). DEHP exposure revealed statistical significance (OR = 1.16, 95% CI: 1.01–1.33). Subgroup analyses suggested significant heterogeneity in MEHHP (I^2^ = 86.6%, *p* = 0.06) and MEOHP (I^2^ = 81.5%, *p* = 0.02) exposure in the Asian population (Table S12).

A total of nine studies have confirmed that postnatal exposure to PAEs has been shown to significantly increase the risk of rhinitis (OR = 1.07, 95% CI: 1.04–1.11) (Figure S8). Of the 13 PAE metabolites, MCOP (OR = 1.14), MEHHP (OR = 1.17), MEOHP (OR = 1.15), MECPP (OR = 1.13), and DEHP (OR = 1.20) demonstrated a specific dose–effect relationship (lower limit of the 95% CI ≥ 1.00). Subgroup analyses revealed that geography, child age, and study design were unable to account for the observed heterogeneity (Table S13).

**Figure 8:**
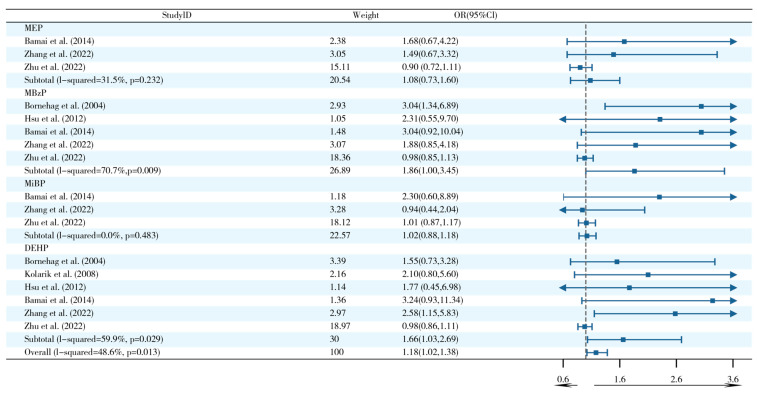
Forest plots of postnatal PAE exposure from indoor dust and rhinitis [1,20,25,35,44,64].

Six studies revealed that exposure to PAEs from indoor dust sources was significantly associated with the risk of rhinitis (OR = 1.18, 95% CI: 1.02–1.38) (Figure 8). Specificity analysis of four PAE metabolites (including MEP, MBzP, etc.) revealed a prominent risk effect of MBzP (OR = 1.86, 95% CI: 1.00–3.45) with DEHP (OR = 1.66, 95% CI: 1.03–2.69) exposure. Subgroup analyses suggested that the heterogeneity for MBzP stemmed from the case–control study design (I^2^ = 78.7%, *p* = 0.009), whereas the heterogeneity for DEHP involved both Asian populations (I^2^ = 67.8%, *p* = 0.026) and case–control studies (I^2^ = 65.5%, *p* = 0.034) (Table S14).

**Figure 9:**
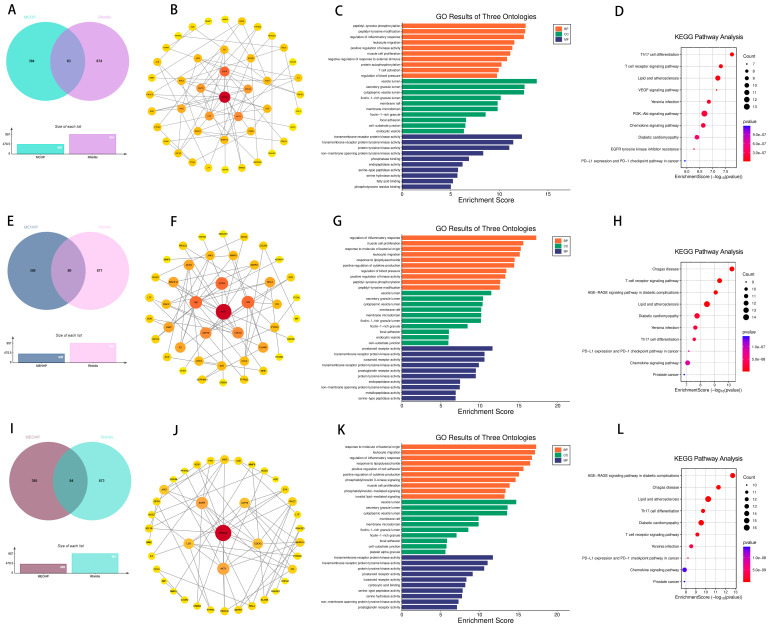
Venn diagram of potential targets of toxicity of MCOP to rhinitis (**A**); PPI of potential targets of toxicity of MCOP to rhinitis (**B**); GO (**C**) and KEGG (**D**) enrichment analyses of potential targets of toxicity of MCOP to rhinitis. Venn diagram of potential targets of toxicity of MEHHP to rhinitis (**E**); PPI of potential targets of toxicity of MEHHP to rhinitis (**F**); GO (**G**) and KEGG (**H**) enrichment analyses of potential targets of toxicity of MEHHP to rhinitis. Venn diagram of potential targets of toxicity of MECPP to rhinitis (**I**); PPI of potential targets of toxicity of MECPP to rhinitis (**J**); GO (**K**) and KEGG (**L**) enrichment analyses of potential targets of toxicity of MECPP to rhinitis.

**Figure 10:**
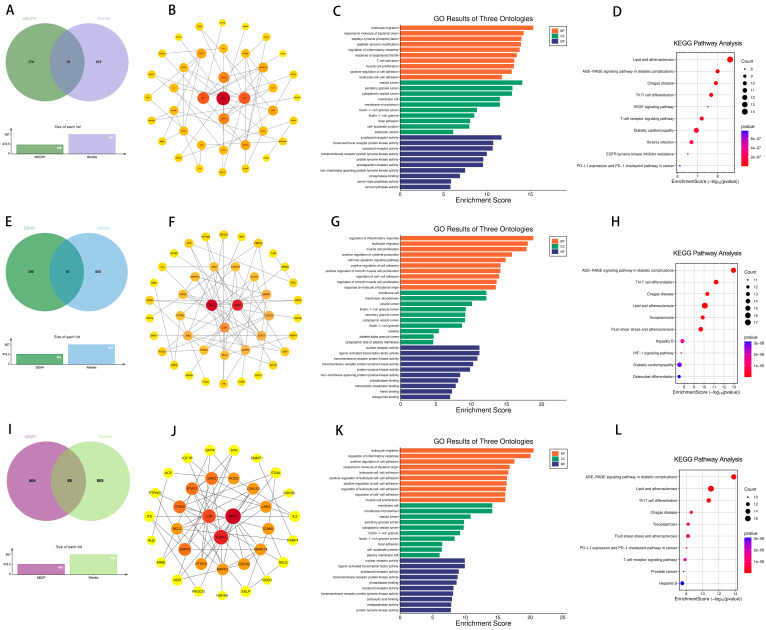
Venn diagram of potential targets of toxicity of DEHP to rhinitis (**A**); PPI of potential targets of toxicity of DEHP to rhinitis (**B**); GO (**C**) and KEGG (**D**) enrichment analyses of potential targets of toxicity of DEHP to rhinitis. Venn diagram of potential targets of toxicity of MEOHP to rhinitis (**E**); PPI of potential targets of toxicity of MEOHP to rhinitis (**F**); GO (**G**) and KEGG (**H**) enrichment analyses of potential targets of toxicity of MEOHP to rhinitis. Venn diagram of potential targets of toxicity of MBzP to rhinitis (**I**); PPI of potential targets of toxicity of MBzP to rhinitis (**J**); GO (**K**) and KEGG (**L**) enrichment analyses of potential targets of toxicity of MBzP to rhinitis.

**Figure 11:**
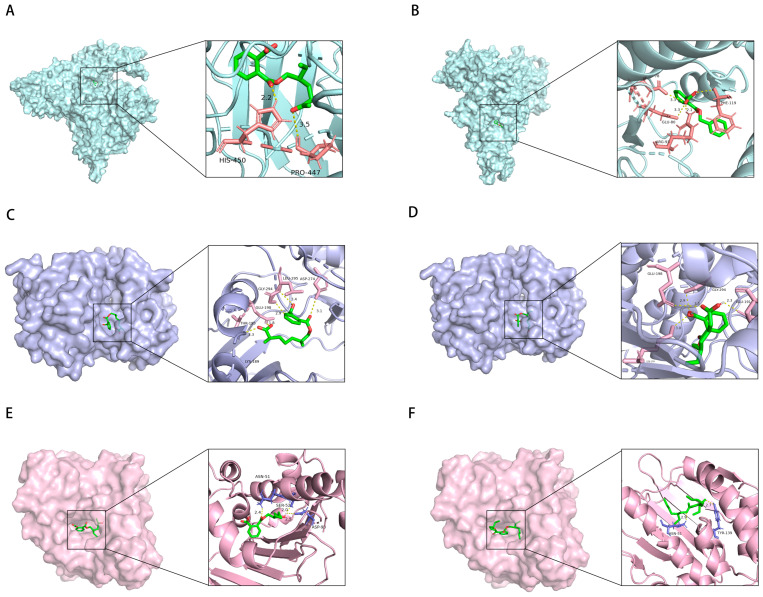
Molecular docking result of MBzP and PIK3CA (**A**); molecular docking result of MEOHP and PIK3CA (**B**); molecular docking result of MCOP and AKT1 (**C**); molecular docking result of MECPP and AKT1 (**D**); molecular docking result of DEHP and HSP90AA1 (**E**); molecular docking result of MEHHP and HSP90AA1 (**F**).

The intersection of disease genes with the targets of DEHP, MBZP, MCOP, MECPP, MEHHP, and MEOHP generated 97, 88, 83, 78, 80, and 84 shared genes, respectively (Figure 9 and Figure 10). These targets showed strong associations with PAE-induced allergic rhinitis. Key targets of DEHP, MBZP, MCOP, MECPP, MEHHP, and MEOHP, such as EGFR, AKT1, and PIK3CD, were identified. Notably, different types of PAEs shared common core targets, and both GO and KEGG analyses revealed overlapping enriched pathways, with the most prominent being the AGE-RAGE signaling pathway in diabetic complications, atherosclerosis, Th17 cell differentiation, and the T cell receptor signaling pathway. Subsequently, molecular docking analyses were conducted to investigate the interactions between PAEs and these core targets (Table 1). The docking results were visualized in 3D representations using PyMOL 3.0.5, respectively (Figure 11).

### 2.7. Sensitivity Analysis and Publication Bias

When one study was excluded from the pooled estimate, the resulting data remained largely unchanged, indicating that the results were relatively robust (Figures S9–S19). Publication bias between PAE exposure and allergic diseases was assessed using Begg’s and Egger’s tests (Figure S20). The funnel plot results demonstrated that most studies fell within the 95% confidence interval, suggesting that publication bias was not a significant factor in the studies examining the association between PAE exposure and allergic diseases. Moreover, the E-value of positive results was calculated to evaluate the potential influence of unmeasured confounding effects on causal conclusions (Table S15).

## 3. Discussion

Environmental PAE exposure may threaten health [67]. In recent years, a growing number of studies have reported that PAE exposure interferes with the immune system of children, leading to allergic diseases [68,69]. In the current study, the effect of prenatal and postnatal PAE exposure on allergic diseases such as wheezing, asthma, eczema, and rhinitis in children was analyzed separately. Our results indicated that prenatal exposure to PAEs may be associated with an increased risk of wheezing and eczema, postnatal PAE exposure could raise the risk of wheezing, eczema, and rhinitis, and postnatal exposure to PAEs from indoor dust could raise the risk of wheezing, eczema, and rhinitis.

The study results indicated that postnatal exposure to MEHHP and MEOHP is associated with an increased risk of wheezing. This finding is consistent with results from studies conducted on mouse models and asthma cohorts [30]. Postnatal exposure to DEHP from indoor dust was related to elevated asthma risk in our study. Bornehag et al. examined the concentration of PAEs in indoor dust and found that DEHP in dust was positively associated with doctor-diagnosed asthma in children [25]. Multiple studies have failed to find a significant correlation between prenatal and postnatal PAEs and childhood wheezing and asthma [10,29]. The results obtained in this study indicate that most classes of PAEs were not associated with wheezing and asthma in children. However, the discrepancies observed in various studies may be attributed to factors such as the duration of sample storage, the age of the children, the different methodologies employed for assessing respiratory conditions, and the age and number of participants involved.

In our study, several types of prenatal PAE exposure were associated with childhood eczema. A US cohort study found that prenatal exposure to MBzP was associated with eczema in early childhood. It was found that MBzP can affect the skin lesions by decreasing methylation of the thymic stromal lymphopoietin gene [70]. However, prenatal exposure to MBzP also had the same results in our study. In addition, we also found postnatal MCOP, MEOHP, and DEHP exposure were associated with eczema risk. A study based on Korean children also found a correlation between these PAEs from children’s urine and the risk of childhood eczema [46].

Numerous investigations have elaborated on the link between environmental factors such as green spaces, air pollution, PAEs, and rhinitis. The cohort study from China found prenatal PAE exposure associated with a significantly increased risk of rhinitis in male infants. Hwang et al. similarly investigated differences about the correlation between exposure to PAEs and allergic disease in children of different genders [36].

In the network toxicology study of this study, it was also shown that there is indeed a non-negligible association between PAEs and allergic diseases and that there may be a commonality in the way different types of PAEs affect allergic diseases, with the key protein targets being SRC, AKT1, HSP90AA1, PIK3CA, LCR, and others. The proteins SRC, AKT1, HSP90AA1, PIK3CA, and LCR are critically implicated in allergic pathogenesis through diverse mechanistic roles. SRC, a non-receptor tyrosine kinase, regulates immune cell activation and inflammatory signaling by modulating T-cell receptor and cytokine pathways [71,72,73]. AKT1, a central node in the PI3K/AKT/mTOR axis, drives eosinophil survival, mast cell degranulation, and Th2 cytokine production, amplifying allergic inflammation. Previous studies also have demonstrated that DEHP can induce cell apoptosis, with one of the primary mechanisms being the inhibition of the PI3K/Akt/Bcl-2 signaling pathway via oxidative damage [72]. HSP90AA1 stabilizes client proteins (e.g., glucocorticoid receptors and NF-κB components), thereby fine-tuning immune responses and corticosteroid sensitivity [74]. PIK3CA, encoding the catalytic subunit of PI3K, activates downstream AKT signaling to promote cell proliferation and survival in epithelial and immune cells, exacerbating barrier dysfunction and hypersensitivity. LCK drives T-cell receptor signaling to promote Th2 polarization and cytokine release (e.g., IL-4 and IL-13), directly exacerbating IgE-mediated allergic inflammation in diseases such as asthma and atopic dermatitis. Collectively, these molecules orchestrate allergic cascades via inflammation amplification, immune hyperactivation, and tissue remodeling, positioning them as potential therapeutic targets for conditions like atopic dermatitis and asthma.

The GO and KEGG results also suggest that the pathways by which PAEs affect allergic diseases are focused on cytokines and inflammatory responses. Similar studies had indicated that PAEs have been shown to affect the immune cells activated to Th2 immunity and cause the increased number of IL-4 and IL-13 cytokines [75]. Prenatal PAEs have also been found to cause increased Th2 immune cells in offspring through epigenetic modification pathways [67]. In an animal experiment, DEHP caused a significant reduction in the number of CD8α dendritic cells (DCs) and bone marrow DC progenitors in the spleen of mice by affecting dendritic cell homeostasis, differentiation, and peroxisome proliferator-activated receptor gamma (PPARγ) activity, which ultimately led to allergic asthma [76]. Wang B et al. reported that prenatal exposure to PAEs can activate the T cell immune-related JAK-STAT signaling pathway, resulting in an increase in eosinophils and the production of IL-4 and IL-13 cytokines [69]. Although there are studies linking other proteins to asthma, direct associations are lacking. Current studies on the mechanisms of PAE-induced allergic diseases are mainly focused on inflammation and epigenetic modifications, and there was an urgent need for research into other mechanisms to fill this gap.

The main advantage of this study is that we proved the effect of prenatal PAE exposure and childhood internal and external PAE exposures on allergic diseases and provided the most comprehensive analysis about the link between the class of PAEs and allergic diseases in children. Different gestational periods were also grouped to determine the effect of the window of prenatal exposure on childhood allergic disease when studying prenatal exposures. However, there are some limitations for this study. Firstly, the number of studies included in this study is still limited. Secondly, the self- or parent-reported information was used for most of the disorders rather than diagnoses from clinicians. Therefore, recall and memory bias may exist. Finally, we did not conduct experimental studies to validate the potential therapeutic pathways analyzed.

## 4. Materials and Methods

### 4.1. Search Strategy

A total of 4847 studies were reviewed using PubMed and Web of Science to investigate the relationship between prenatal and postnatal exposure to PAEs and the occurrence of allergic diseases in children, up until May 2023. Ultimately, 54 citations were included in this meta-analysis. The detailed search strategy can be found in Table S2.

### 4.2. Inclusion and Exclusion Criteria

We included the studies if they fulfilled the following criteria: (1) cohort, case–control, and cross-sectional studies; (2) children were the participants; (3) participants were observed to have PAE exposure; (4) serum, urine, and indoor dust were employed as measured samples; (5) studies that reported at least one allergic disease, such as eczema, rhinitis, asthma, and wheezing; and (6) studies that reported OR and 95% CI. Criteria for exclusion were as follows: (1) studies involving replicated data; (2) studies lacking risk estimates; (3) studies involving animals; (4) reviews; and (5) letters. Two authors conducted the screening and evaluation of articles independently, consulting with a third author in cases of uncertainty. Disagreements were resolved through discussion to determine the literature for inclusion.

### 4.3. Data Extraction and Quality Assessment

In the present study, two authors were responsible for independently collating the following data: publication year, author, country, period, study method, sample size, biological specimen sampling results, outcomes of PAEs, odds ratios (ORs), relative risks (RRs) or hazard ratios (HRs) with 95% confidence intervals (CIs), and information regarding adjustments.

The quality of the included literature was evaluated and assigned a score using the Newcastle–Ottawa (NOS) quality assessment scale, which encompasses three domains: selection of participants, comparability, and exposure/outcome. The overall score of a case–control or cohort study is 9 points, and a cross-sectional study is 10 points. A score of 7–9 for case–control or cohort studies and 8–10 for cross-sectional studies indicates high-quality research. A score below 4 demonstrates that the study is a low-quality research study.

### 4.4. Statistical Analysis

STATA statistical software (version 12.0; College Station, TX, USA) was used to analyze data. A random effects model was employed to calculate overall pooled effect estimates and to examine the correlation between PAEs and allergic diseases. The sources of heterogeneity were explored by subgroup analysis according to prenatal exposure period, study design, study area, and children’s age. In sensitivity analyses, we employed a study-by-study exclusion to ascertain its impact on the overall estimate. The presence of publication bias was discerned through the utilization of the Egger regression and funnel plot. The threshold for statistical significance was set at *p* < 0.05, with a two-sided approach employed.

### 4.5. Prediction of Potential Targets of Toxicity of PAEs to Allergic Diseases

Gather the structure and SMILES characters from PubChem (https://pubchem.ncbi.nlm.nih.gov, accessed on 1 March 2025). Candidate targets of PAEs were predicted by the SwissTargetPrediction (http://swisstargetprediction.ch, accessed on 1 March 2025), TargetNet (http://targetnet.scbdd.com, accessed on 1 March 2025), and Pharmmapper (https://lilab-ecust.cn/pharmmapper/index.html, accessed on 1 March 2025). Additionally, the Gene Cards database (https://www.genecards.org, accessed on 5 March 2025) and the Online Mendelian Inheritance (OMIM) database (https://www.omim.org, accessed on 5 March 2025) were employed to retrieve potential therapeutic targets related to allergic diseases. The redundancy of the candidate targets was eliminated. The name of these targets was standardized in the Uniprot database (https://www.uniprot.org, accessed on 7 March 2025).

### 4.6. Construction of Network and Enrichment Analysis of Potential Targets

The STRING database (https://cn.string-db.org, accessed on 15 March 2025) was used to construct a target-to-target of candidate targets. The confidence score of protein interactions was set as >0.9. A network of molecular pathways was constructed using Cytoscape_v3.10.3 using general data. A set of hub genes was selected based on the degree according to CytoHubba of Cytoscape software. Enrichment analysis was performed using the clusterProfiler and ClueGO packages in R for pathway and functional annotation. 

### 4.7. Molecular Docking

The potential binding between the three chemical components of plasticizers and core targets was explored using molecular docking techniques. AutoDock Vina v1.2.x was used for the docking process and PyMOL 3.0.5 for visualization.

## 5. Conclusions

Our study sought to ascertain the impact of maternal exposure to PAEs on the occurrence of adverse pregnancy outcomes. The results showed that prenatal PAEs exposure could increase the risk of wheezing and eczema in children, postnatal PAEs exposure could raise the risk of wheezing, eczema, and rhinitis in children, and postnatal exposure to PAEs from indoor dust could raise the risk of wheezing, eczema, and rhinitis in children. Overall, such endeavors will aid in confirming the associations between prenatal and postnatal PAE exposure and childhood allergic diseases, furnishing policymakers with evidence for the regulation of PAEs and their potential implications on children’s health. Given the current limitations of available research, it is imperative to conduct more extensive cohort and mechanism-related studies to gain a deeper understanding of the relationship.

## Figures and Tables

**Table 1:** Molecular docking results.

PAEs	PubChem CID	Target	PDB ID	Vina Score
DEHP	8343	HSP90AA1	1UYK	−8
		AKT1	3MV5	−6.9
		SRC	1YOM	−6.4
		EGFR	1M14	−6
		LCK	20F4	−5.5
MBzP	31736	PIK3CA	2RD0	−8.9
		LCK	20F4	−8.3
		SRC	1YOM	−6.9
		PIK3R1	3HHM	−6.9
		EGFR	1M14	−6.7
		AKT1	3MV5	−6.4
MCOP	486427123	AKT1	3MV5	−7.3
		PIK3CD	5T8F	−7.2
		EGFR	1M14	−6.8
MEHHP	170295	HSP90AA1	1UYK	−7.9
		AKT1	3MV5	−7.8
		LCK	20F4	−7.5
		EGFR	1M14	−6.7
		SRC	1YOM	−6.4
MEOHP	119096	PIK3CA	2RD0	−7.7
		AKT1	3MV5	−7.3
		PIK3CD	5T8F	−7.1
		SRC	1YOM	−6.6
		EGFR	1M14	−6.3
MECPP	148386	AKT1	3MV5	−7.5
		LCK	20F4	−7.4
		EGFR	1M14	−6.2

## Data Availability

The original contributions presented in this study are included in the article/Supplementary Materials. Further inquiries can be directed to the corresponding author(s).

## Supplementary Materials

The supporting information can be downloaded at: https://www.mdpi.com/article/10.3390/ijms26136103/s1.
