# Supplementary material for: Maternal Phthalate Exposure and Allergic Diseases in Children: A Meta-Analysis and Network Toxicology
Source: Int J Mol Sci. 2025 Jun 25;26(13):6103. doi: 10.3390/ijms26136103 (PMC12250265; doi:10.3390/ijms26136103)
Supplement: Supplementary file 1 [file ijms-26-06103-s001.zip › Supplementary Figure.pdf]

Maternal Phthalate Exposure and Allergic Diseases in Children: A Meta-Analysis and Network Toxicology

Yi Xiang, Yanming Lv, Wenhao Fu, Jie Wen, Baixiang Li and Xueting Li *

Department of Hygienic Toxicology, School of Public Health, Harbin Medical University, 157 Baojian Road, Harbin 150081, China
* Correspondence: lxting@hrbmu.edu.cn

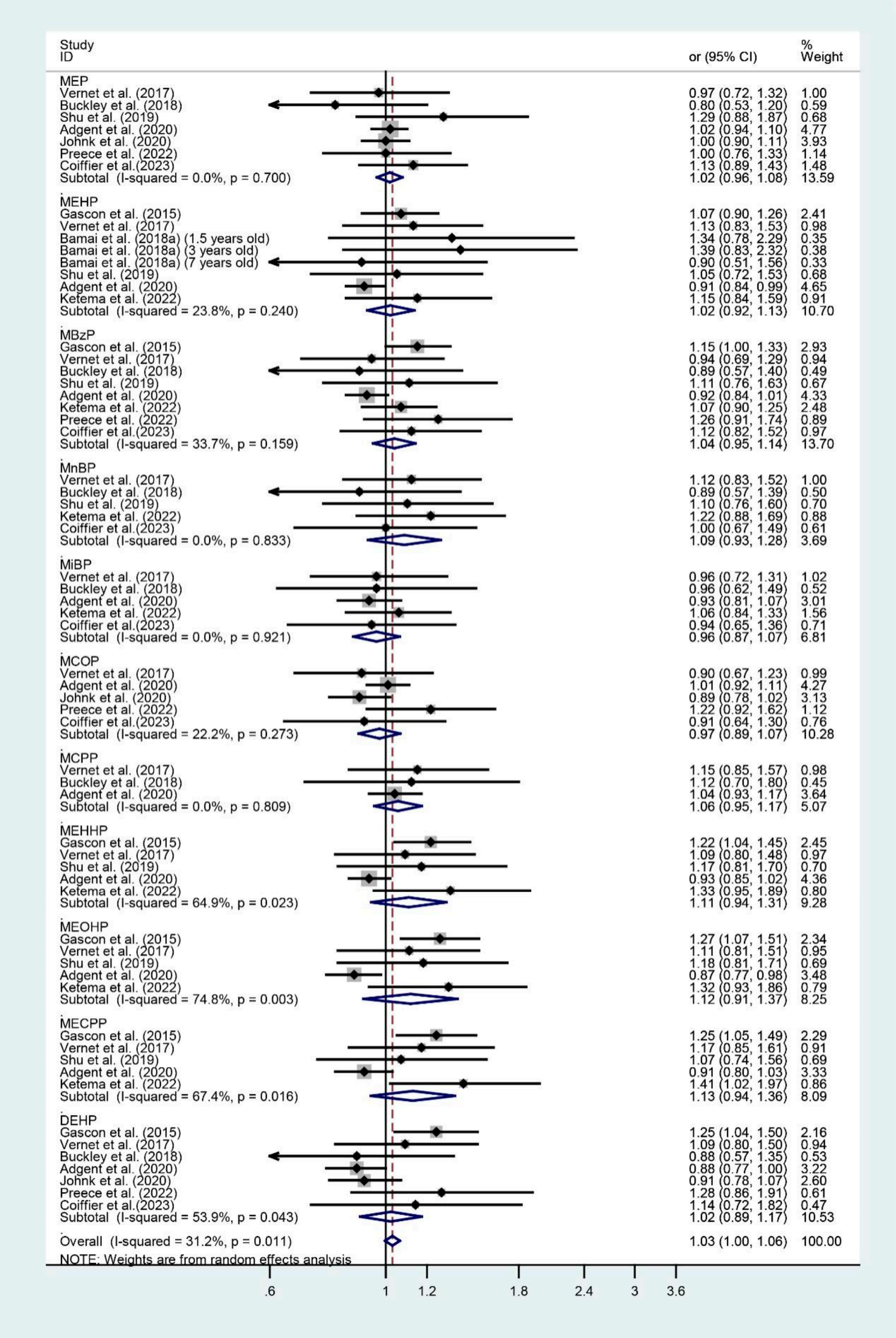

Supplementary Figure S1. Forest plots of prenatal PAEs exposure and childhood wheeze.

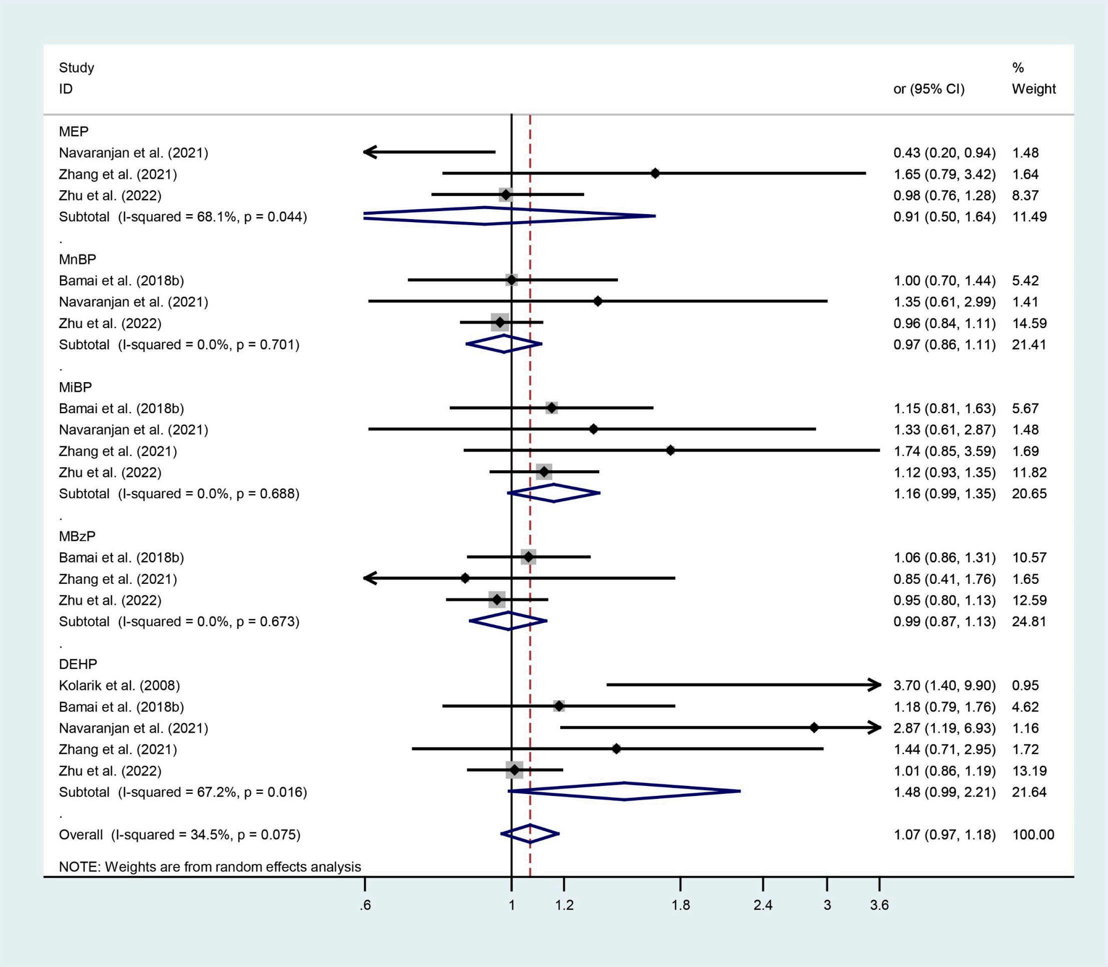

Supplementary Figure S2. Forest plots of postnatal PAEs exposure to PAEs from indoor dust and wheeze.

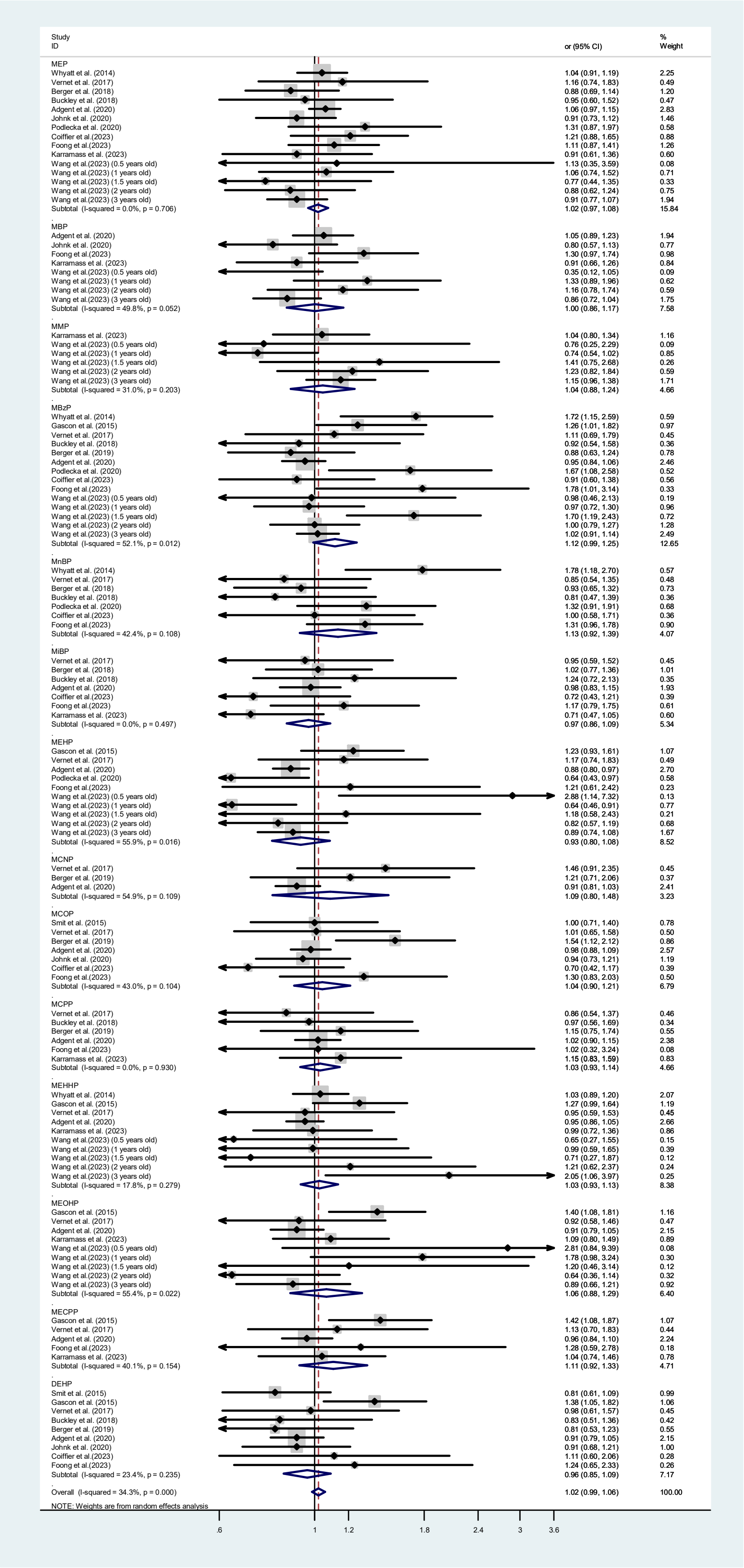

Supplementary Figure S3. Forest plots of prenatal PAEs exposure and children asthma.

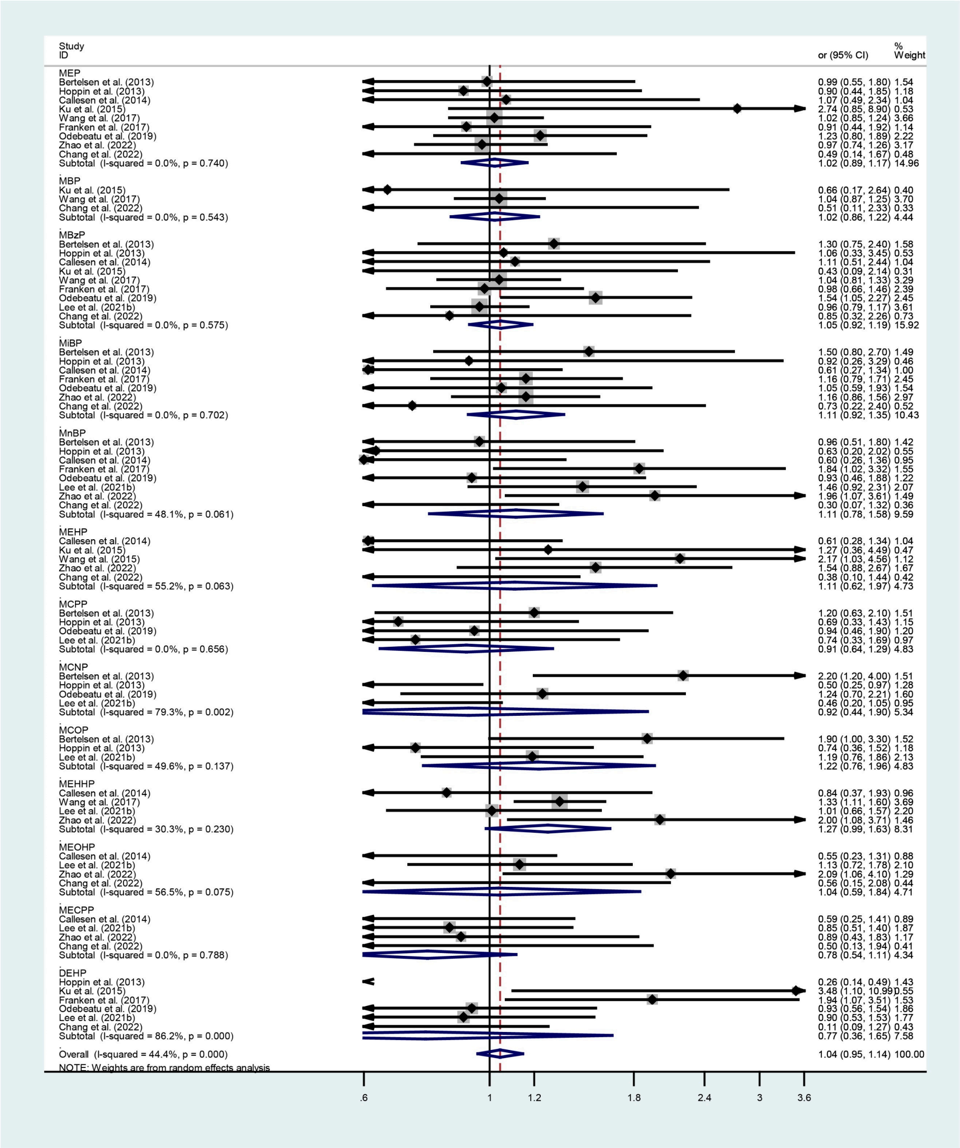

Supplementary Figure S4. Forest plots of postnatal PAEs exposure and asthma.

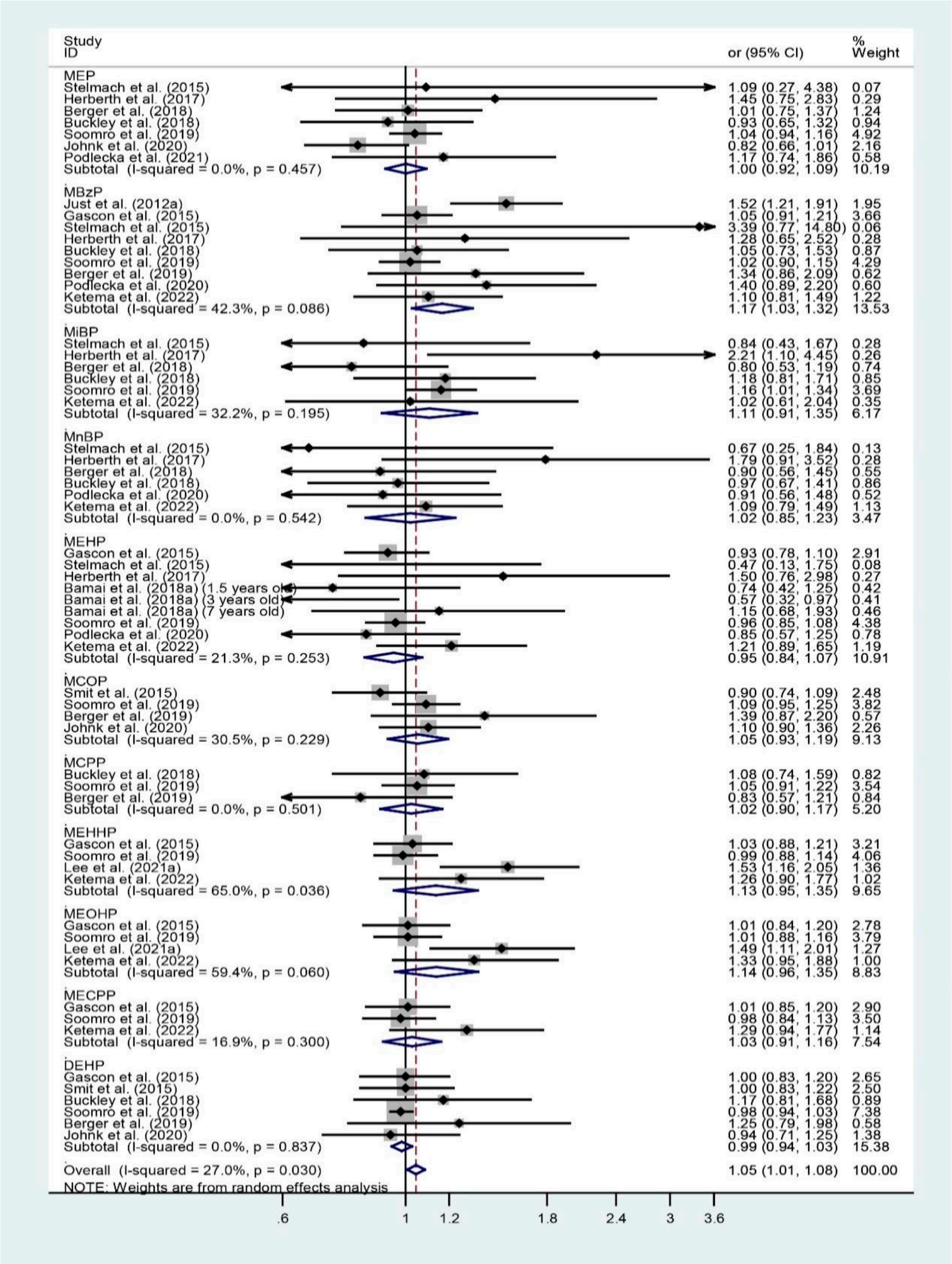

Supplementary Figure S5. Forest plots of prenatal PAEs exposure and childhood eczema.

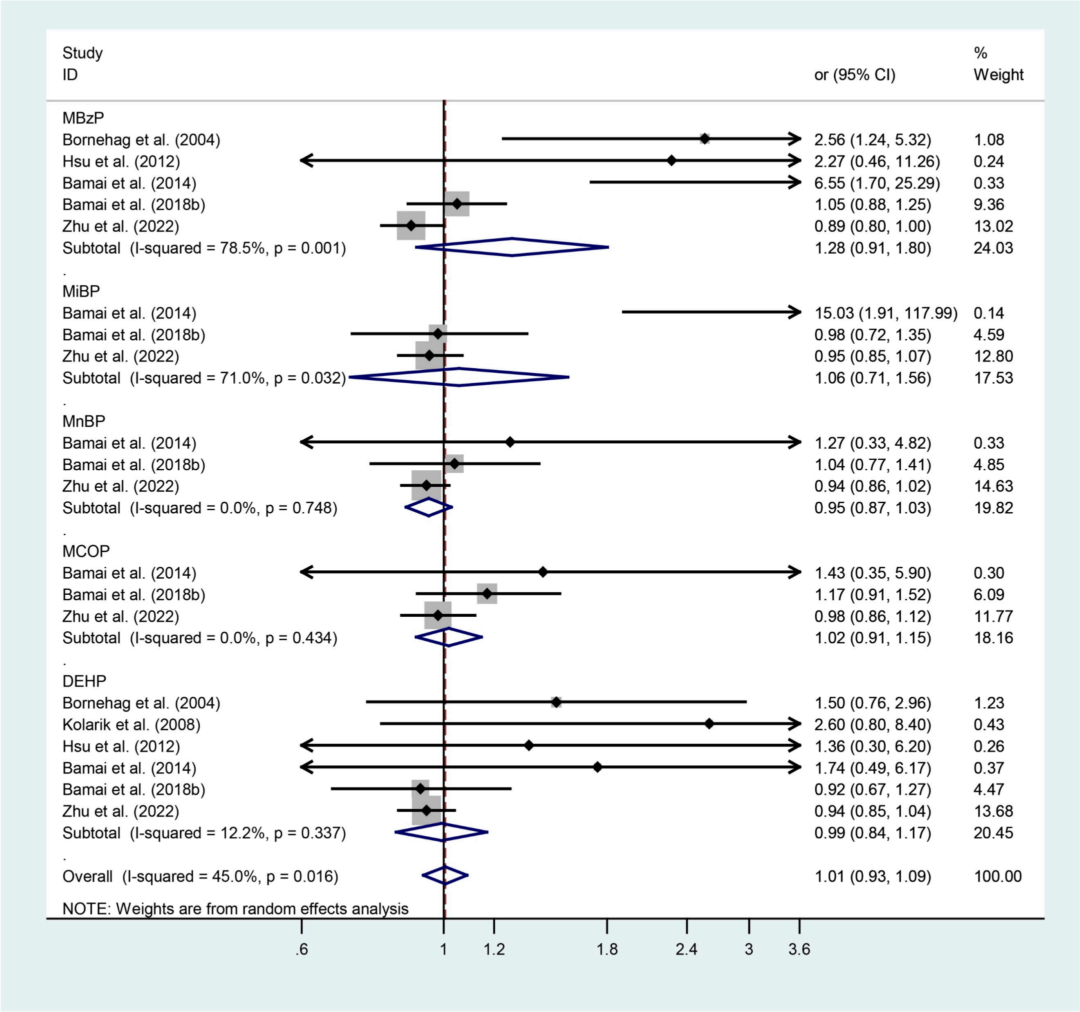

Supplementary Figure S6. Forest plots of postnatal exposure to PAEs from indoor dust and eczema.

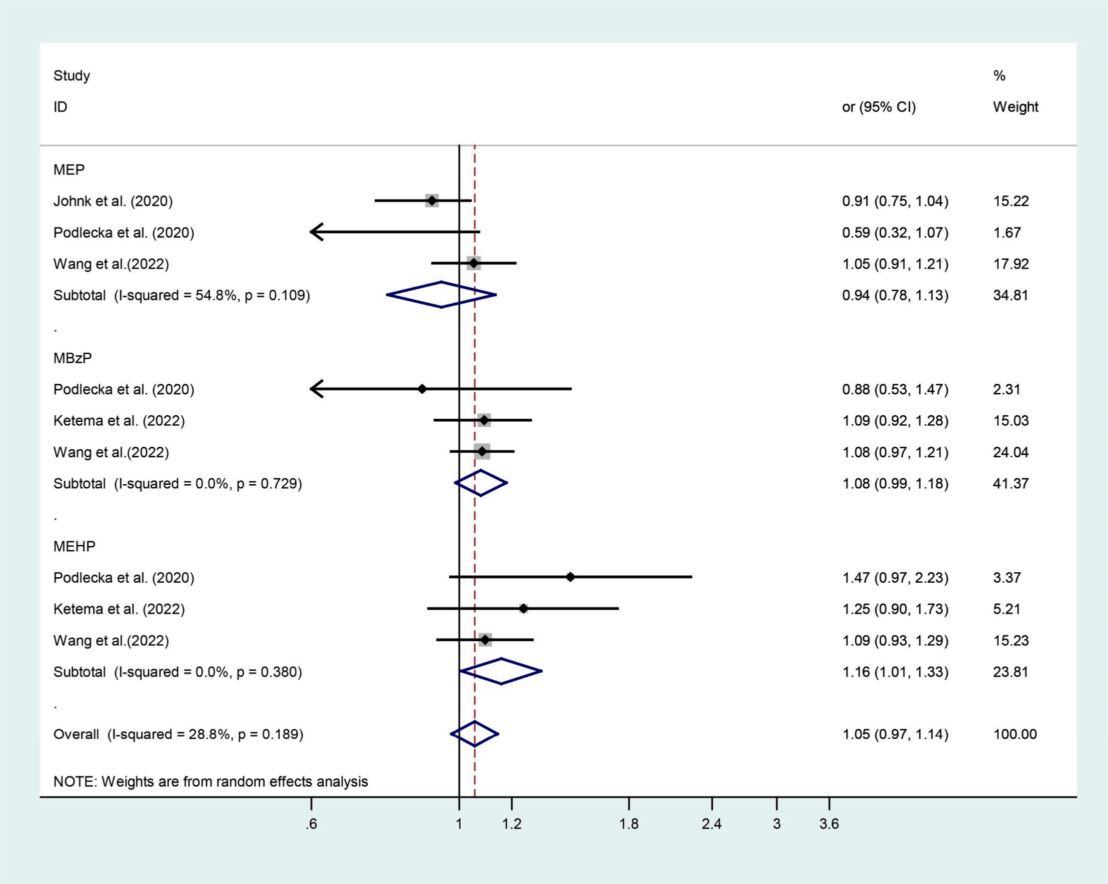

Supplementary Figure S7. Forest plots of prenatal PAEs exposure and rhinitis.

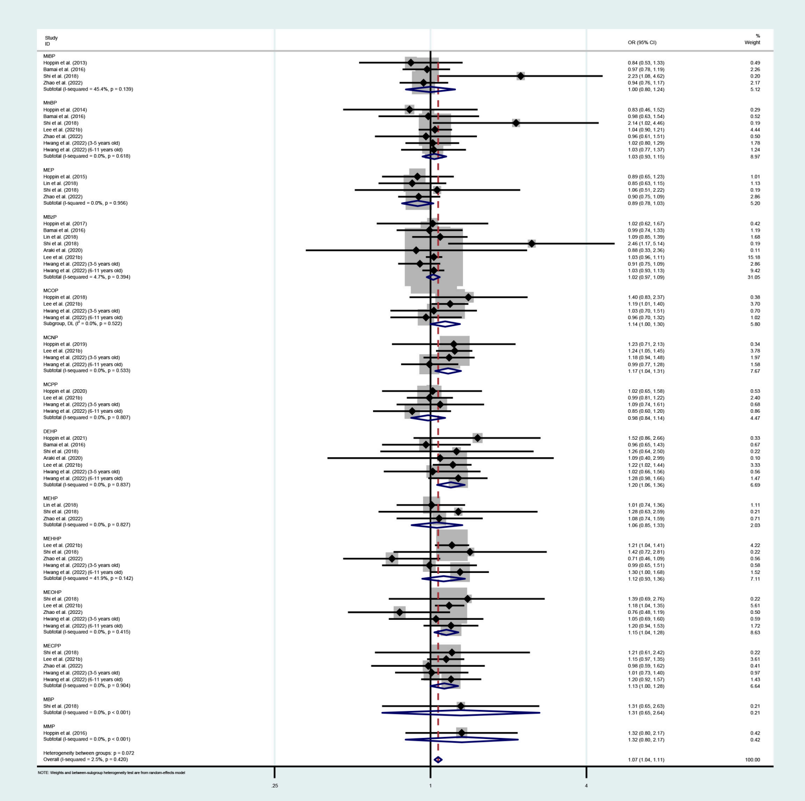

Supplementary Figure S8. Forest plots of postnatal PAEs exposure and rhinitis.

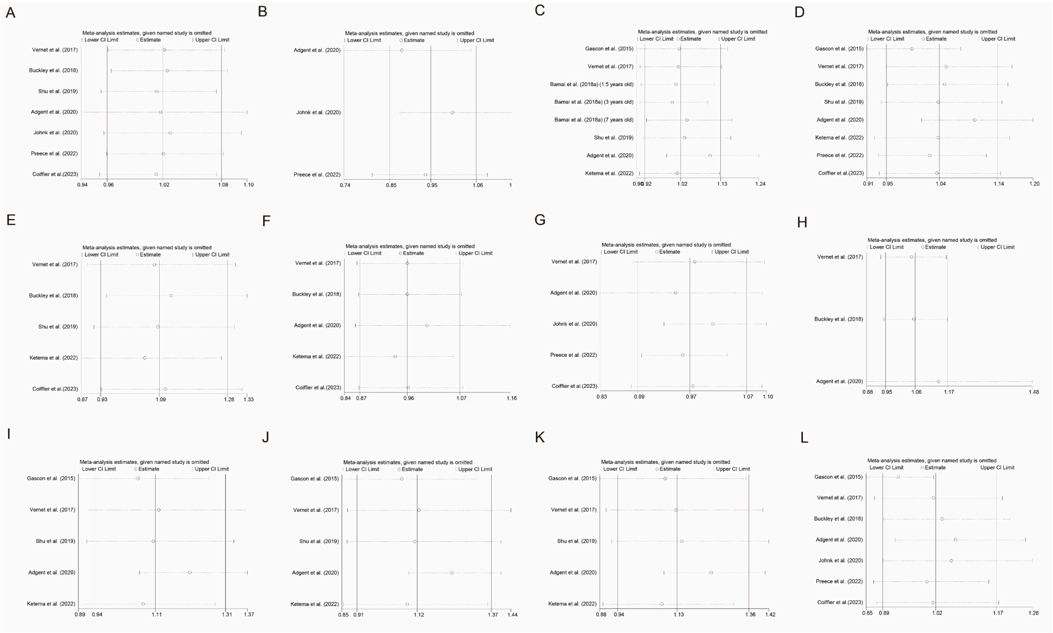

Supplementary Figure S9. Sensitivity analysis of prenatal PAEs exposure and wheeze. (A) MEP; (B) MBP; (C) MEHP; (D) MBzP; (E) MnBP; (F) MiBP; (G) MCOP; (H) MCPP; (I) MEHHP; (J) MEOHP; (K) MECPP; (L) DEHP

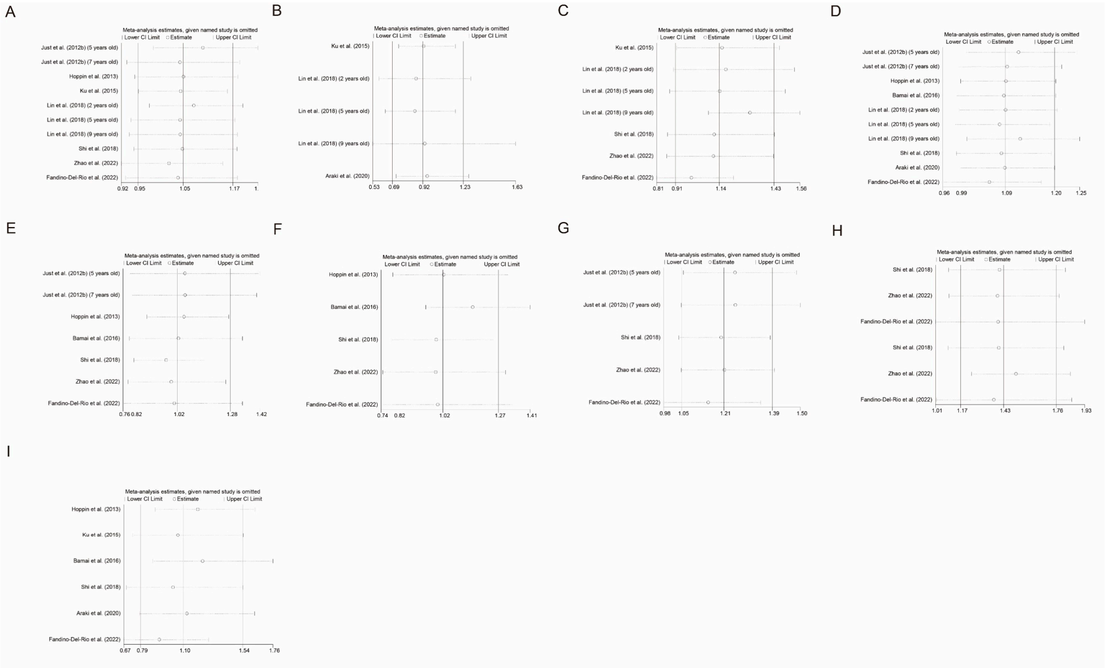

Supplementary Figure S10. Sensitivity analysis of postnatal PAEs exposure and wheeze. (A) MEP; (B) MBP; (C) MEHP; (D) MBzP; (E) MnBP; (F) MiBP; (G) MEHHP; (H) MEOHP; (I) DEHP

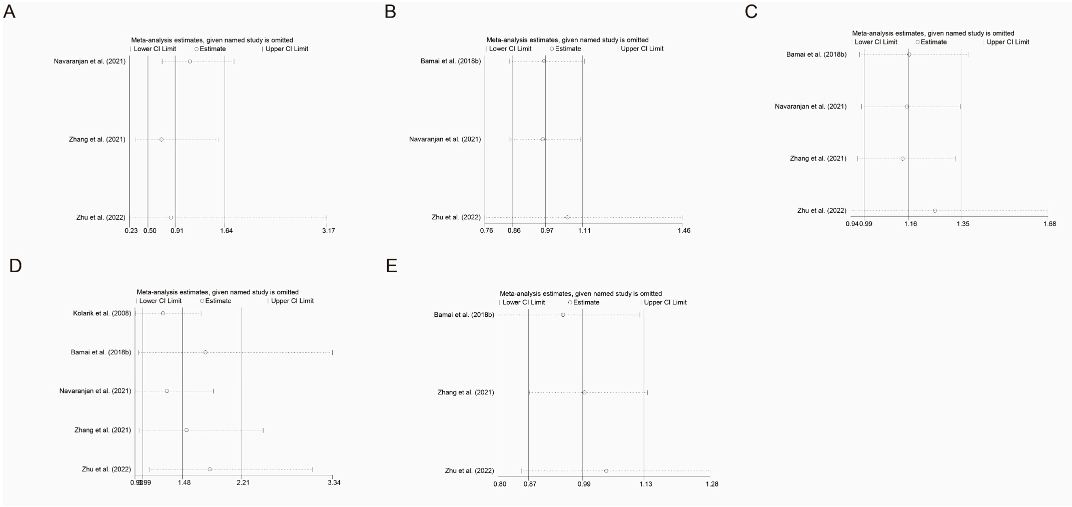

Supplementary Figure S11. Sensitivity analysis of postnatal exposure to PAEs from indoor dust and wheeze. (A) MEP; (B) MnBP; (C) MiBP; (D) MBzP; (E) DEHP

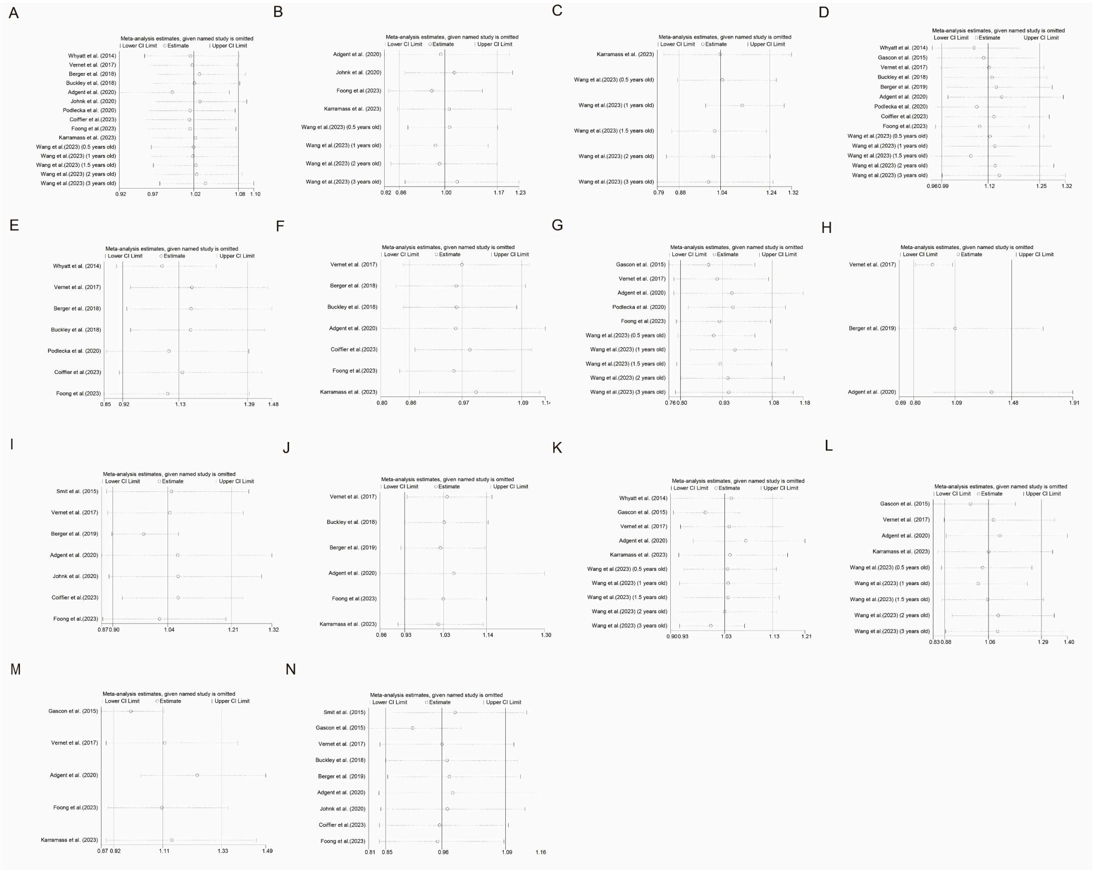

Supplementary Figure S12. Sensitivity analysis of maternal PAEs and asthma. (A) MEP; (B) MBP; (C) MMP; (D) MBzP; (E) MnBP; (F) MiBP; (G) MEHP; (H) MCNP; (I) MCOP; (J) MCPP; (K) MEHHP; (L) MEOHP; (M) MECPP; (N) DEHP

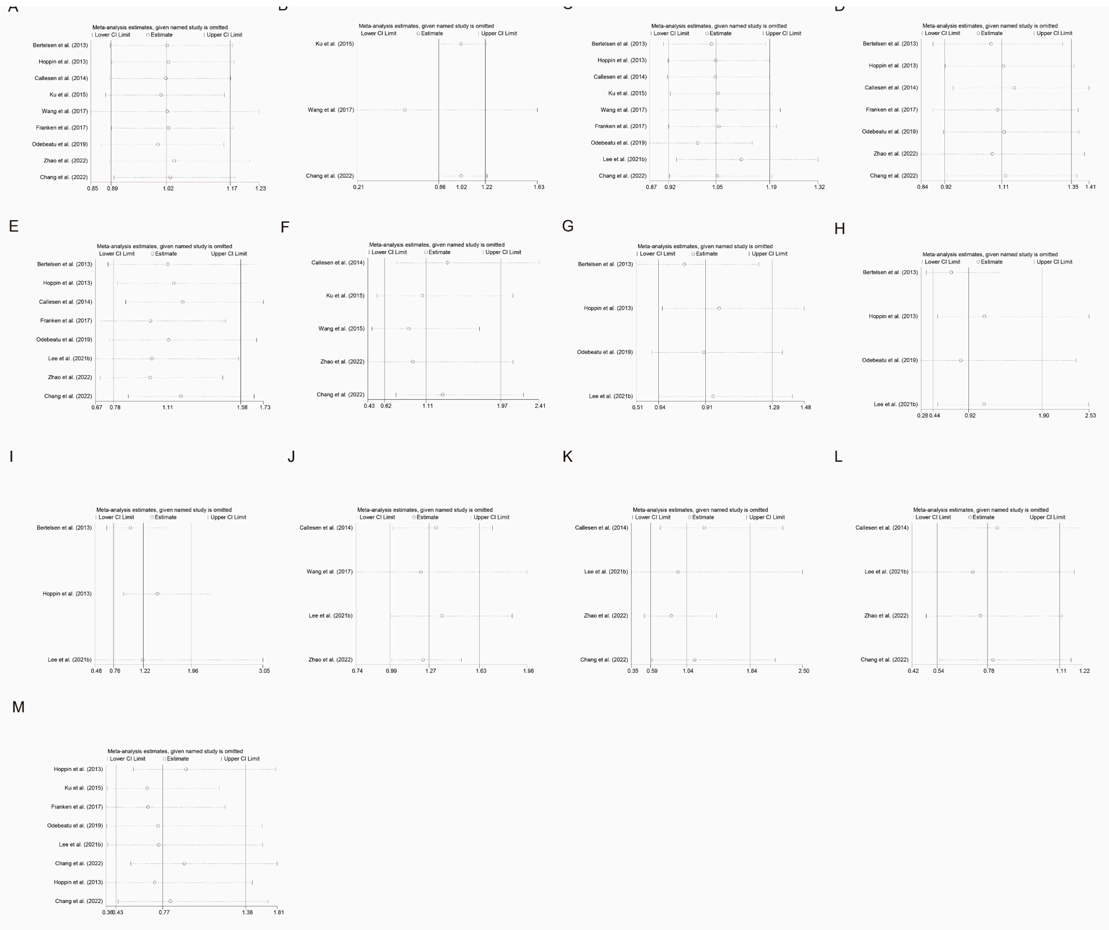

Supplementary Figure S13. Sensitivity analysis of postnatal PAEs exposure and asthma. (A) MEP; (B) MBP; (C) MBzP; (D) MiBP; (E) MnBP; (F) MEHP; (G) MCPP; (H) MCNP; (I) MCOP; (J) MEHHP; (K) MEOHP; (L) MECPP; (M) DEHP

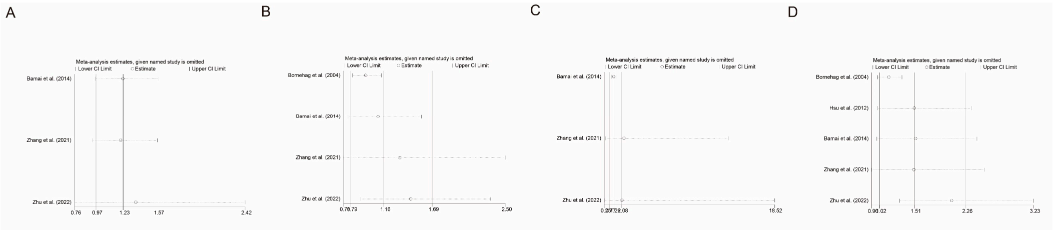

Supplementary Figure S14. Sensitivity analysis of postnatal exposure to PAEs from indoor dust and asthma. (A) MEP; (B) MBzP; (C) MiBP; (D) DEHP.

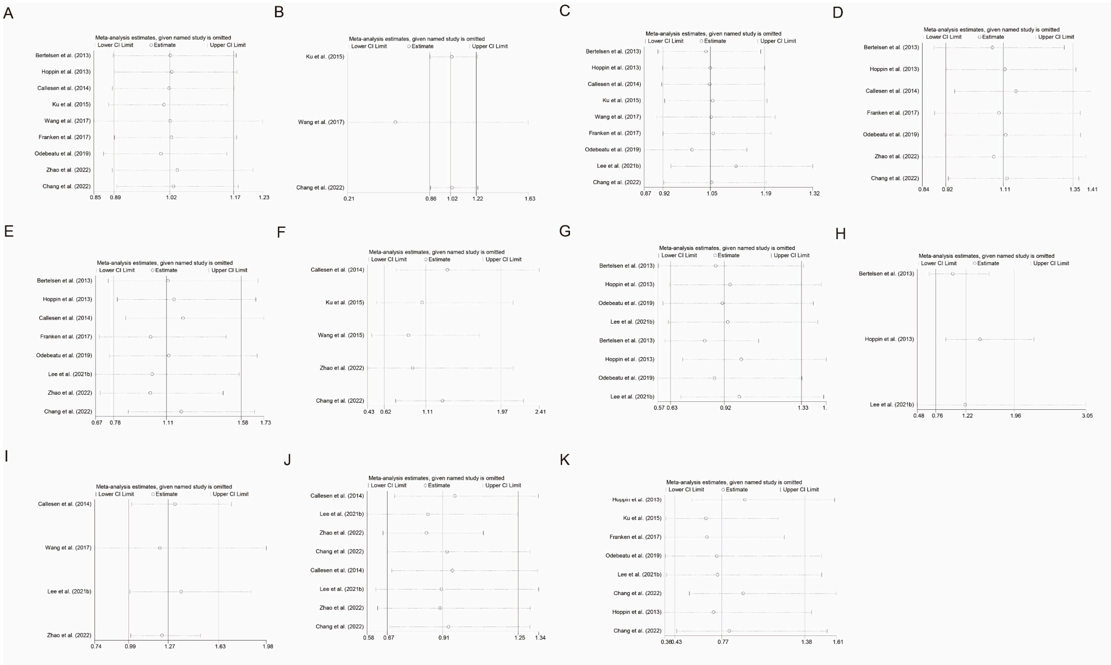

Supplementary Figure S15. Sensitivity analysis of prenatal PAEs exposure and eczema. (A) MEP; (B) MBzP; (C) MiBP; (D) MnBP; (E) MEHP; (F) MCOP; (G) MCPP; (H) MEHHP; (I) MEOHP; (J) MECPP; (K) DEHP

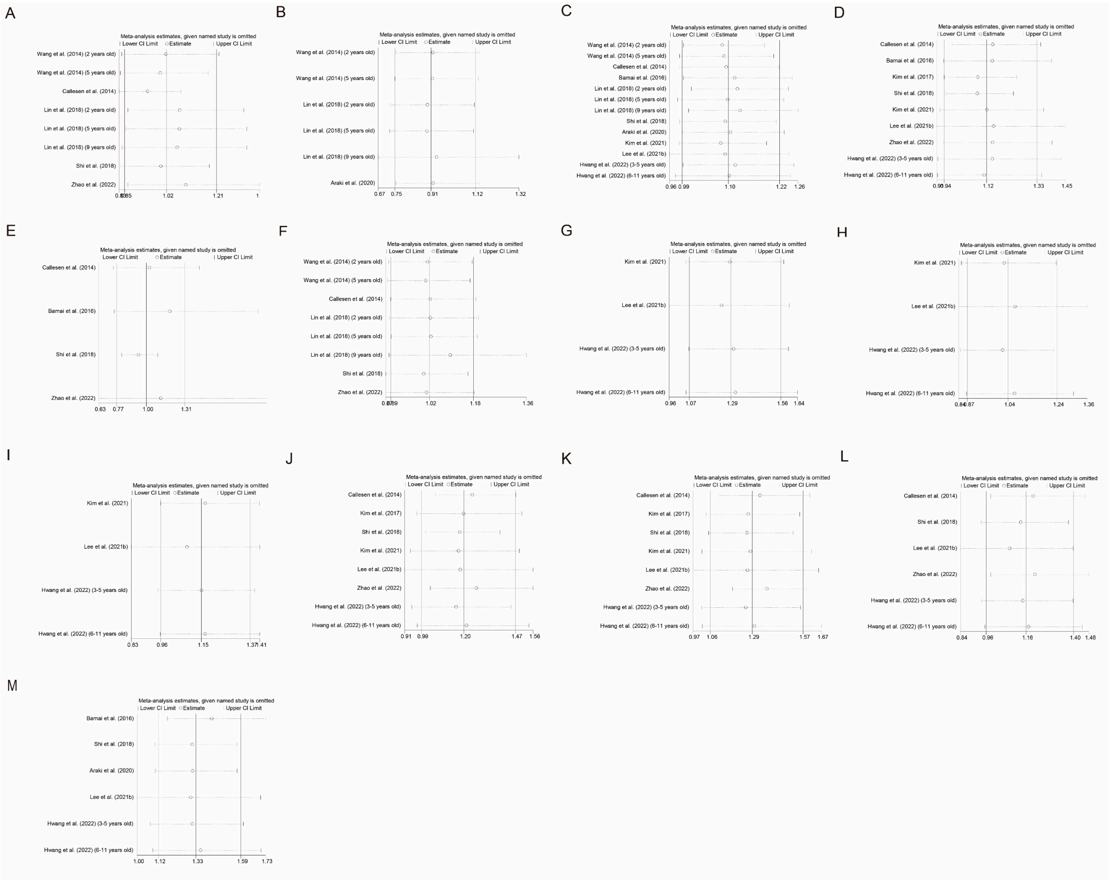

Supplementary Figure S16. Sensitivity analysis of postnatal PAEs exposure and eczema. (A) MEP;(B) MBP; (C) MBzP; (D) MnBP; (E) MiBP; (F) MEHP; (G) MCOP; (H) MCPP; (I) MCNP; (J) MEHHP;

(K) MEOHP; (L) MECPP; (M) DEHP

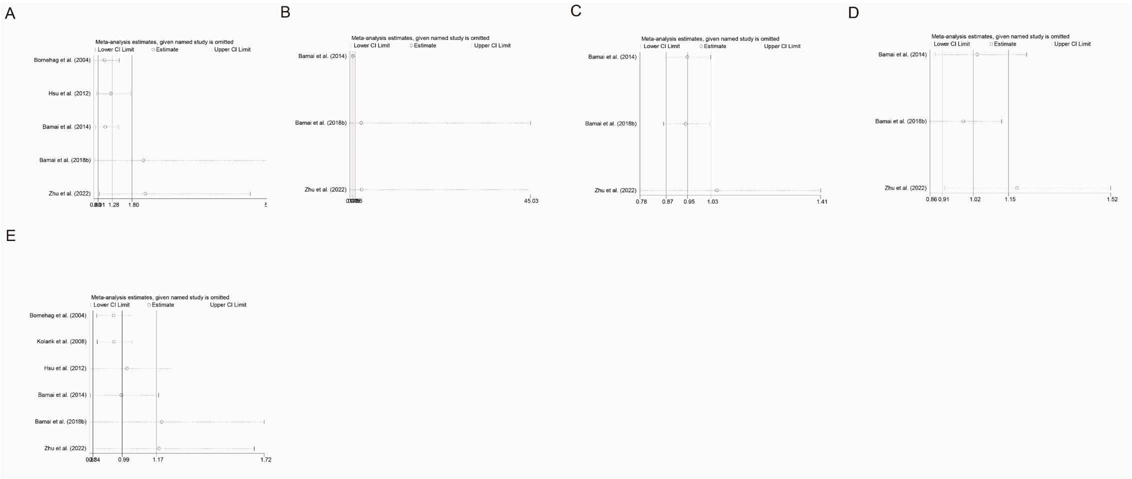

Supplementary Figure S17. Sensitivity analysis of postnatal exposure to PAEs from indoor dust and eczema. (A) MBzP; (B) MiBP; (C) MnBP; (D) MCOP; (E) DEHP

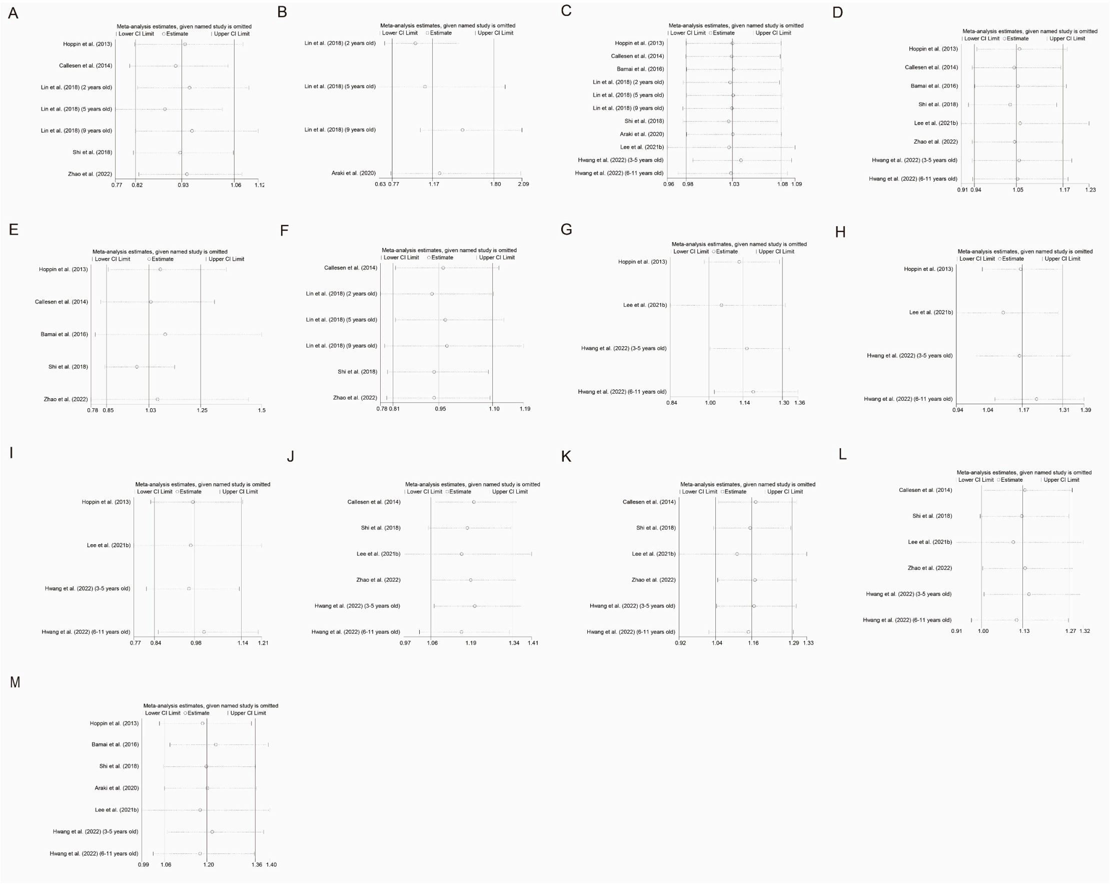

Supplementary Figure S18. (A) MEP;(B) MBP; (C) MBzP; (D) MnBP; (E) MiBP; (F) MEHP; (G) MCOP; (H) MCNP; (I) MCPP; (J) MEHHP; (K) MEOHP; (L) MECPP; (M) DEHP

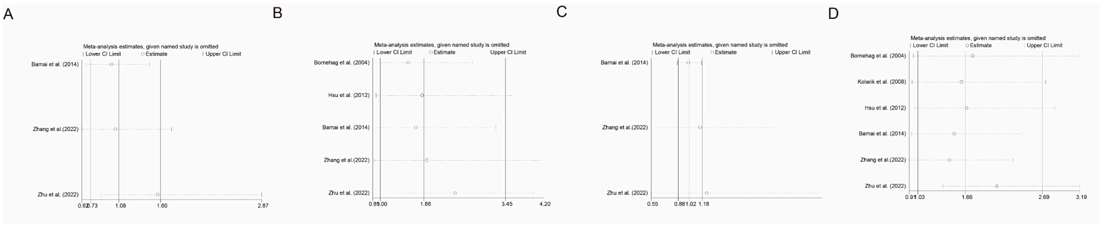

Supplementary Figure S19. Sensitivity analysis of postnatal exposure to PAEs from indoor dust and rhinitis. (A) MEP; (B) MBzP; (C) MiBP; (D) DEHP

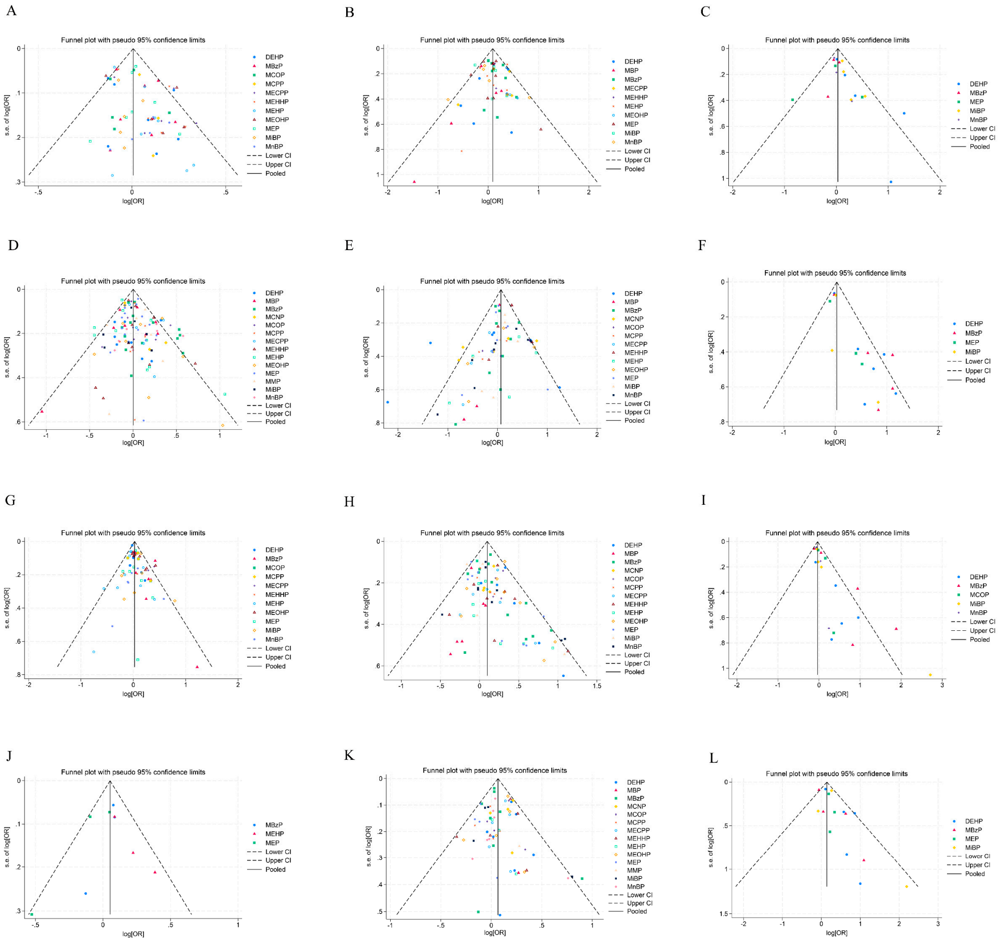

Supplementary Figure S20. (A) Funnel plots of prenatal PAEs exposure and wheezing. (B) Funnel plots of postnatal PAEs exposure and wheezing. (C) Funnel plots of postnatal PAEs exposure from indoor dust and wheezing. (D) Funnel plots of prenatal PAEs exposure and asthma. (E) Funnel plots of postnatal PAEs exposure and asthma. (F) Funnel plots of postnatal PAEs exposure from indoor dust and asthma. (G) Funnel plots of prenatal PAEs exposure and eczema. (H) Funnel plots of postnatal PAEs exposure and eczema. (I) Funnel plots of postnatal PAEs exposure from indoor dust and rhinitis. (J) Funnel plots of prenatal PAEs exposure and rhinitis. (K) Funnel plots of postnatal PAEs exposure and rhinitis. (L) Funnel plots of postnatal PAEs exposure from indoor dust and rhinitis.
