# Supplementary material for: Maternal Phthalate Exposure and Allergic Diseases in Children: A Meta-Analysis and Network Toxicology
Source: Int J Mol Sci. 2025 Jun 25;26(13):6103. doi: 10.3390/ijms26136103 (PMC12250265; doi:10.3390/ijms26136103)
Supplement: Supplementary file 1 [file ijms-26-06103-s001.zip › Supplementary Table.pdf]

Maternal Phthalate Exposure and Allergic Diseases in Children: A Meta-Analysis and Network Toxicology

Yi Xiang, Yanming Lv, Wenhao Fu, Jie Wen, Baixiang Li and Xueting Li *

Department of Hygienic Toxicology, School of Public Health, Harbin Medical University, 157 Baojian Road, Harbin 150081, China
* Correspondence: lxting@hrbmu.edu.cn

Supplementary Table S1. Characteristics of the included studies on the association between phthalates exposure and allergic diseases.

Reference	Location	Study period	Study method	Sample	Children Age (years)	Chemicals	Samples collected	Exposure assessment	Outcomes	Diagnostic method	NOS	Adjusted variables
Bornehag et al. (2004)	Sweden	2001.1-2002.4	Case control	400	3-8	MBzP, DEHP	indoor dust	GC/MSD	asthma, eczema, rhinitis	Questionnaire	6	Sex, age, smoking at home, type of building, construction period, self-reported flooding during preceding 3 years, and the other phthalate variable.
Kolarik et al. (2008)	Bulgaria	2004.4-2004.8	Case control	184	1	DEHP	indoor dust	GC/flame ionization	wheeze, eczema, rhinitis	Questionnaire	6	Age, sex, smoking at home during pregnancy and first year of child's life, current smoking at home, allergy or asthma in family.
Hsu et al. (2012)	China	2005-2006	Cross-sectional study	101	3-9	MBP, MBzP, MEHP, DEHP	indoor dust	GC-MS	asthma, eczema, allergic rhinitis	ISAAC	7	Children gender, age, presence of fever, and if taken any medication in the recorded week, as well as parents smoking status, allergic history and education levels, and the month the sampling took place were all taken into account in multiple logistic regression

Just et al. (2012a)	USA	1999-2006	Cohort study	407	2	MBzP	Maternal urine during third trimester	HPLC-MS/MS	eczema	ISAAC	7	Specific gravity, sex, and race/ethnicity.
Just et al. (2012b)	USA	2006-2010	Cohort study	244	5/7	MEP, MnBP, MBzP, MEHHP	Children's urine	HPLC-MS/MS	wheeze	ISAAC	8	Specific gravity, sex, and race/ethnicity.
Bertelsen et al. (2013)	Norway	1992.1-1993.4	Cross-sectional study	623	10	MEP, MnBP, MiBP, MBzP, MCPP, MCOP, MCNP	Children's urine	HPLC-ESI-MS/MS	asthma	Questionnaire	8	Urine SG, sex, parental asthma, and household income.
Hoppin et al. (2013)	USA	2005-2006	Cross-sectional study	779	6-17	MiBP, MnBP, MEP, MMP, MBzP, MCOP, MCNP, MCPP, DEHP	Children's urine	HPLC-ESI-MS/MS	wheeze, asthma, rhinitis	Questionnaire	7	Age, race, gender, BMI, creatinine, and cotinine.
Callesen et al. (2014)	Denmark	2008.8-2009.4	Cross-sectional study	440	3-5	MEP, MnBP, MiBP, MBzP, MEHP, MEHHP, MEOHP, MECPP	Children's urine	LC-ESI-MS/MS	asthma, eczema, rhino-conjunctivitis	ISAAC	7	Sex, breastfed >3 months, antibiotics, single allergic predisposition, visible mold, visible moisture, condensation >5 cm on the window pane in winter, cat, dog, pet avoidance, changed cleaning habits, smoke inside, social

												class.
Bamai et al. (2014)	Japan	2006.9-2006.12	Cross-sectional study	122	2	MMP, MEP, MiBP, MnBP，MBzP, DEHP, MCOP	indoor dust	GC/MS	asthma, atopic dermatitis, allergic rhinitis	Questionnaire	7	Gender, age strata, environmental tobacco smoke, dampness index, furry pets inside the home.
Wang et al. (2014)	China	2004	Cohort study	483	2/5	MEP, MBP, MBzP, MEHP	Children's urine	UPLC-MS/MS	atopic dermatitis	ISAAC	7	Gender, gestational age, maternal education, maternal history of atopy, and pre-natal ETS exposure.
Whyatt et al. (2014)	USA	1998-2006	Cohort study	300	5-11	MBzP, MnBP, MEP, MEHHP	Maternal urine during third trimester	HPLC-MS/MS	asthma	ISAAC	7	Maternal asthma, household smoke exposure, maternal prenatal BPA, maternal prenatal demoralization, maternal prenatal specific gravity, and child age.
Gascon et al. (2015)	Spanish	2004-2008	Cohort study	657	7	DEHP, MBzP, MEHP, MEHHP, MEOHP, MECPP	Maternal urine during first trimester	LC-MS/MS	wheeze, asthma, eczema	ISAAC	7	Maternal history of asthma/allergy and maternal body mass index.
Ku et al.	China	2000.12-	Cohort study	430	8	MEHP, DEHP, MBzP, MEP, MBP	Children's urine	LC-ESI-MS/MS	wheeze, asthma	ISAAC	8	Parental allergies and family members' smoking status.

(2015)		2001.11										
Smit et al. (2015)	Ukraine and Denmark	2002-2004	Cohort study	1024	5-9	MCOP, DEHP	Maternal serum during entire pregnancy	LC-MS/MS	asthma, eczema	ISAAC	8	Maternal allergy, smoking during pregnancy, educational level, maternal age, child sex, child age at follow-up, gestational age at blood sampling, parity, breastfeeding, and birthweight.
Stelmach et al. (2015)	Poland	2007	Cohort study	144	2-2.5	MEP, MiBP, MnBP, MBzP, MEHP, 7OH-MiNP, MOP	Maternal urine during third trimester	HPLC-MS/MS	atopic dermatitis	ISAAC	8	Atopy in family, father's education, frequency of cleaning, breastfeeding.
Wang et al. (2015)	China	2010	Cohort study	256	3	5OH-MEHP	Children's urine	UPLC-MS/MS	asthma	doctor diagnosis	8	Gender, age, prematurity, maternal history of atopy, maternal education, and environmental tobacco smoke exposure.
Bamai et al. (2016)	Japan	2009.10-2010.11	Cross-sectional study	184	6-12	MiBP, MnBP, MBzP,DEHP	Children's urine	GC/MS	wheeze, atopic dermatitis, rhino-	ISAAC	7	Gender, age, and parental history of allergies.

									conjunctivitis			
Wang et al. (2017)	China	2010	Case control	453	3	MEP, MBP, MBzP, MEHHP	Children's urine	UPLC-MS/MS	asthma	ISAAC	7	Urine creatinine, age, gender, maternal history of atopy, maternal education, and environmental tobacco smoke exposure.
Franken et al. (2017)	Denmark	2008.5-2009.7; 2013.3-2013.12	Cross-sectional study	418	14-15	DEHP, MnBP, MBzP, MEP, MiBP	Children's urine	UPLC-MS/MS	asthma	ISAAC	7	gender, age, smoking status and familial asthma.
Herberth et al. (2017)	Germany	2006.3-2008.12	Cohort study	610	3	MEP, MiBP, MnBP, MBzP, MEHP	Maternal blood during third trimester	LC-MS/MS	atopic dermatitis	Questionnaire	6	Sex, maternal atopic dermatitis, maternal smoking, and/or ETS exposure at home, siblings, maternal education, cat ownership, and breast-feeding until 6 months.
Kim et al. (2017)	Korea	2009.5-2010.4	Cross-sectional study	18	3-7	MEHHP, MEOHP, MnBP	Children's urine	HPLC-MS/MS	atopic dermatitis	A diary about atopic dermatitis symptoms	6	Temperature, humidity, and season.

Vernet et al. (2017)	French	2003.4-2006.3	Cohort study	587	5	MEP, MnBP, MiBP, MCPP, MBzP, MCNP, MCOP, MEHHP, MEOHP, MECPP, MEHP, DEHP	Maternal blood during second and third trimester	HPLC-MS/MS	wheeze, asthma	ISAAC	7	Center, residence area, parental history of asthma or allergies, maternal ethnicity, maximal parental education level, maternal or passive smoking during pregnancy, postnatal passive smoking, older siblings, and child care.
Bamai et al. (2018a)	Japan	2002.7-2005.10	Cohort study	314	1.5/3.5/7	MEHP	Maternal blood during second and third trimester	GC/MS	wheeze, eczema	ISAAC	8	Sex, history of maternal allergies, maternal age at delivery, parity, maternal smoking, day care attendance, and household income.
Bamai et al. (2018b)	Japan	2013	Cross-sectional study	296	7	MiBP, MnBP, MBzP, DEHP, MCOP	indoor dust	GC–MS	wheeze, eczema	ISAAC	8	Sex, household income, maternal smoking, parental history of atopy, and FLG mutation.
Berger et al. (2018)	USA	1999-2000	Cohort study	392	7	MEP, MnBP, MiBP	Maternal urine during second and third	HPLC-MS/MS	asthma, eczema	ISAAC	7	Maternal age, parity, season of birth, household income as a proportion of poverty at baseline, child's family history of asthma, active and passive

							trimester					smoking during pregnancy, furry pets in the home during pregnancy, housing density during pregnancy.
Buckley et al. (2018)	USA	1998-2002	Cohort study	165	6 and 7	MEP, MnBP, MiBP, MCPP, MBzP, DEHP	Maternal urine during third trimester	HPLC-MS/MS	wheeze, asthma, eczema	ISAAC	7	Creatinine, maternal age, race/ethnicity, pre-pregnancy body mass index, education, marital status, type of home ownership, smoking during pregnancy, person in household with asthma, person in household with allergies, number of occupants in the home, pets in the home, age at follow-up, and, for overall models, child's sex.
Lin et al. (2018)	China	2004.4-2005.1	Cohort study	470	2/5/9	MEP, MBP, MBzP, MEHP	Children's urine	UPLC–MS/MS	wheeze, atopic dermatitis, allergic rhinitis	ISAAC	8	Children sex, height, body mass index, history of environmental tobacco smoke exposure, incensing at home, and family income.
Shi et al.	China	2011-2012;	Cross-section	434	5-10	MMP, MEP, MiBP, MnBP,MEHHP,	Children's urine	HPLC-MS/MS	wheeze, eczema,	ISAAC	8	Gender, age, BMI, breastfeeding time, family smoking exposure,

(2018)		2013-2014	al study			MECPP, MEOHP, MEHP, MBzP, DEHP			rhinitis			residence area, maternal education, annual household income, history of parental asthma, wall materials in children's bedrooms and floor materials in children's bedrooms.
Shu et al. (2019)	Sweden	2007-2010	Cohort study	1062	1	MEP, MnBP, MBzP, MEHP, MEHHP, MEOHP, MECPP, MCMHP, MHiNP, MOiNP, MCiOP, MHiDP, MCiNP, MOiNCH	Maternal urine during first trimester	LC-MS/MS	wheeze	ISAAC	8	Sex, mother's education, mother's age, asthma in the family, smoking and creatinine in urine.
Soomro et al. (2019)	France	2003-2006	Cohort study	604	5	MEP, MBP, MiBP, MECPP, MEHHP, MEOHP, MEHP, MBzP, MCOP, MCPP, MCNP, DEHP	Maternal urine during second and third trimester	HPLC-ESI-MS/MS	eczema	ISAAC	7	Parental asthma/rhinitis/eczema, maternal smoking, maternal age, maternal BMI, maternal education level, gestational age, number of siblings, and recruitment center.
Berger et al. (2019)	USA	1999-2008	Cohort study	392	7	MBzP, MCNP, MCOP, MCPP, DEHP	Maternal urine during	HPLC-MS/MS	asthma, eczema	ISAAC	7	Maternal age, parity, household income as a proportion of poverty at baseline, child's

							entire pregnancy					family history of asthma, maternal education.
Odebeatu et al. (2019)	USA	2007-2008; 2009-2010, 2011-2012	Cross-sectional study	7765	6-17	MEP, MiBP, MnBP, MBzP, MCNP, MCPP, DEHP	Children's urine	HPLC-ESI-MS/MS	asthma	Questionnaire	7	Age, sex, ethnicity/race, waist circumference, cotinine, poverty and urinary creatinine.
Adgent et al. (2020)	USA	2007-2011	Cohort study	1481	4-6	MEP, MBP, MiBP, MBzP, MCPP, MCOP, MCNP, DEHP, MEHP, MEHHP, MEOHP, MECPP	Maternal urine during third trimester	HPLC-MS/MS	wheeze, asthma	ISAAC	6	Maternal age, maternal race, maternal ethnicity, maternal education, prenatal smoking, prepregnancy BMI, maternal history of asthma, child sex, year of child's birth and study site.
Araki et al. (2020)	Japan	2008-2010	Cohort study	128	7-12	DBP，MBzP，DEHP	Children's urine	GC-MS/MS	wheeze, eczema, rhinitis	ISAAC	6	Sex, grade, annual income and dampness index.
Johnk et al. (2020)	Denmark	2010-2012	Cohort study	552	5	MEP, MBP, DEHP, MCOP	Maternal urine during second and third	LC-MS/MS	wheeze, asthma, eczema, rhinitis	ISAAC	6	Maternal age, maternal education, parity and family history of asthma/allergy.

							trimester					
Podlecka et al. (2020)	Poland	2007	Cohort study	145	9	MBzP, MnBP, MEHP, MEP	Maternal urine during third trimester	HPLC-MS/MS	asthma, atopic dermatitis, allergic rhinitis	ISAAC	7	Sex, parental atopy, smoking in the study participants' domicile, keeping domestic animals, skin prick test results, spirometry, and levels of the remaining phthalates.
Kim et al. (2021)	Korea	2015-2017	Cross-sectional study	797	12-17	MEHHP, MEOHP, MECCP, MnBP, MBzP, MCOP, MCNP, MCPP	Children's urine	UPLC-ESI-MS/MS	atopic dermatitis	ISAAC	7	Gender, school grade, household income, secondhand smoke, and BMI.
Lee et al. (2021a)	Korea	2006-2012	Cohort study	413	0.5	MEHHP, MEOHP	Maternal urine during entire pregnancy	HPLC-MS/MS	atopic dermatitis	Questionnaire	8	Urinary creatinine concentrations corresponding to the stage of pregnancy, mother's characteristics (age, social economics status, education levels, pre-pregnancy BMI, parity, history of atopic dermatitis, urinary cotinine level corresponding to the stage of pregnancy), and infant' sex.
Lee et al.	Korea	2015-2017	Cross-section	2208	3-17	MnBP, MBzP, MCOP, MCNP,	Children's urine	UPLC-ESI-	asthma, atopic	ISAAC	8	Age, sex, household income, maternal and paternal education

(2021b)			al study			MCPP, DEHP, MEHHP, MEOHP, MECPP		MS/MS	dermatitis, allergic rhinitis			levels, body mass index, urinary creatinine level, and urinary cotinine level.
Navaranjan et al. (2021)	Canada	2008-2012	Case control	897	2-5	MEP, MiBP, MNBP, MZBP, DEHP	indoor dust	GC-MS	wheeze	Questionnaire	7	Study site, sex, parental history of asthma and household income.
Zhang et al. (2021)	China	2011.4-2012.4; 2013.3-2014.12	Case control	266		MMP, MEP, MiBP, MBP, MNOP, MNP, DEHP, MBzP	indoor dust	GC-MS	wheeze, asthma	ISAAC	7	Children’s gender, age, family atopic history, family members’ smoking, residence location, early renovation status, and indoor dampness.
Zhao et al. (2022)	China	2013.5-2014.12; 2013.9-2016.1	Cohort study	243	4-8	MEP, MiBP, MnBP, MEHP, MECPP, MEHHP, MEOHP	Children's urine	HPLC-MS	wheeze, asthma, eczema, rhinitis	ISAAC	8	Age, gender, allergic history, home dampness, environmental tobacco smoke exposure and measurement season.
Chang et al. (2022)	China	2010	Case control	201	3-18	MMP, MEP, MiBP, MnBP, MEHP, MEHHP, MEOHP, MECPP, MCMHP, DEHP, MBP, MBzP, MiNP	Children's urine	LC-ESI-MS/MS	asthma	ISAAC	8	Creatinine, passive smoking during pregnancy, annual income, and raising a furry or feathery pet.

Fandino-Del-Rio et al. (2022)	USA	2007.4-2009.6	Cohort study	148	5-17	DEHP, MECPP, MEHHP, MEHP, MEOHP, MBzP, MCINP, MCIOP, MCPP, MECPTP, MEHHTP, MEP, MIBP, MnBP	Children's urine	LC-MS/MS	wheeze	ISAAC	8	Age, sex, race, caregiver education, season, and presence of smokers in the home as a proxy for environmental tobacco smoke exposure.
Hwang et al. (2022)	Korea	2015-2017	Cross-sectional study	1458	3-5; 6-11	DEHP, MEHHP. MEOHP, MECPP, MBzP, MnBP, MCOP, MCNP, MCPP	Children's urine	UPLC-ESI-MS/MS	atopic dermatitis, allergic rhinitis	ISAAC	8	Age, BMI, monthly household income and status of passive smoking.
Ketema et al. (2022)	Japan	2003-201; 2012-2017	Cohort study	386	7	MiBP, MnBP, MBzP, MEHP, MEOHP, MEHHP, MECPP, MiNP	Maternal urine during first trimester	UPLC-MS/MS	wheeze, eczema, rhino-conjunctivitis	ISAAC	8	Sex, parental history of allergy, creatinine.
Preece et al. (2022)	Sweden	2007-2010	Cohort study	1148	2	MEP, MBP, MBzP, DEHP, MCOP, MHiDP, MCiNP, MOiNCH	Maternal urine during first trimester	LC-MS/MS	wheeze	ISAAC	8	Child sex, parental asthma/rhinitis, mother's education, smoking, and creatinine in urine.
Wang	China	2013.5-	Cohort	234	0-3	MMP, MEP, MBP,	Maternal	HPLC-	allergic	Questionna	7	Maternal age, race, residence in

et al. (2022)		2014.9	study	8		MBzP, MEHP, MEOHP, MEHHP	urine during first trimester	MS/MS	rhinitis	ire		the previous 6 months, smoking status, alcohol consumption.
Zhang et al. (2022)	China	2013.3-2014.12	Case control	266		MMP, MEP, MiBP,MBP, DEHP, MNOP,MBzP,MNP	indoor dust	GC-MS	allergic rhinitis	ISAAC	7	Children's gender, age, family atopic history, environment tobacco smoke, residence location, early renovation condition, early antibiotics exposure, and indoor dampness.
Zhu et al. (2022)	China	2013.9-2016.1	Case control	398	0-8	MEP,MiBP, MnBP,MBzP, MCOP, DEHP	indoor dust	GC-MS	wheeze, asthma, eczema, rhinitis	ISAAC	7	Age, gender, family allergic history, environmental tobacco smoke exposure, dampness and measurement seasons.
Coiffier et al. (2023)	French	2014-2017	Cohort study	484	3	MEP, MnBP, MiBP, MBzP, DEHP, MCOP	Maternal urine during second and third trimester	HPLC-MS/MS	wheeze, asthma	ISAAC	6	Child sex, tobacco exposure, weight at 3 years, parents' highest diploma and antecedents of rhinitis, maternal age and BMI before pregnancy, parity, season of urine sampling and breastfeeding duration.
Foong et al.	Australia	1989-1991	Cohort study	846	5 and 13	MBP, MiBP, MnBP, MEP, MHBP,	Maternal serum	LC/MS	asthma	Questionnaire	7	Household income, maternal smoking, breastfeeding status

(2023)						MBzP, MCPP, MiDP, MEHP, MECPP, DEHP, MCOP	during second and third trimester					and maternal age.
Karramass et al. (2023)	Netherlands	2004.2-2005.7	Cohort study	1020	13	MMP, MEP, MBP, MIBP, MCPP, MBzBP, MECPP, MEHHP, MEOHP, MCMHP, MHpP, MHxP	Maternal urine during entire pregnancy	HPLC-ESI-MS/MS	asthma	ISAAC	6	Confounders including parity, ethnicity, education, folic acid use periconceptional, smoking during pregnancy, pre-pregnancy BMI and Diet Quality score.
Wang et al. (2023)	China	2013.5-2014.9	Cohort study	3267	0.5/1/1.5/2/3	MMP, MEP, MBP, MBzP, MEHP, MEOHP, MEHHP	Maternal urine during first trimester	HPLC-MS/MS	asthma	Questionnaire and doctor's diagnosis	8	Pre-pregnancy BMI, residence in the previous 6 months in 12-month girl and baby drug allergy in 18-month girl and pre-pregnancy BMI in 24-month girl and baby drug allergy in 36-month girl for first trimester.

Supplementary Table S2. The strategies used for searching about phthalates and allergic diseases.

#1 (Mono(2-ethylhexyl) phthalate) OR (mono-isobutyl phthalate) OR (mono-ethyl phthalate) OR (Mono(2-ethyl-5-oxohexyl) phthalate) OR (Mono(2-ethyl-5-hydroxyhexyl) phthalate) OR (Mono(2-ethyl-5-carboxypentyl) phthalate) OR (Mono(3-carboxypropyl) phthalate) OR (Mono(carboxyisooctyl) phthalate) OR (Mono(carboxyisononyl) phthalate) OR (Monobenzyl phthalate) OR (Mono(n-butyl) phthalate) OR (MEHP) OR (MiBP) OR (MEP) OR (MEOHP) OR (MEHHP) OR (MECPP) OR (MCPP) OR (MCOP) OR (MCNP) OR (MBzP) OR (MBP) OR (phthalates) OR (phthalate)

#2 ((((((((((allergy) OR (atopy)) OR (sensitization)) OR (eczema)) OR (dermatitis)) OR (rhinitis)) OR (wheeze)) OR (asthma)) OR (allergic rhinitis)) OR (rhinoconjunctivitis)) OR (allergic disease)
#3 #1 AND #2

Supplementary Table S3. Subgroup analysis results on prenatal PAEs exposure and risk of wheeze.

**Class of PAEs**	**Subgroup**	**No.**	**Subgroup analysis**	**Heterogeneity**	
			**OR (95% CI)**	**I^2 (%)**	***p*-Value**
**MEP**	**Timing of specimen collection**				
	First trimester	1	1.29(0.89,1.88)		
	Second and third trimester	6	1.01(0.96,1.07)	0.00%	0.81
	**Study area**				
	Europe	4	1.01(0.92,1.11)	0.00%	0.631
	North America	3	1.02(0.94,1.11)	3.50%	0.355
	**Children age (years old)**				
	<3	1	1.29(0.89,1.88)		
	≥3	6	1.01(0.96,1.07)	0.00%	0.81
**MBP**	**Study area**				
	North America	1	1.00(0.87,1.15)		
	Europe	2	0.88(0.74,1.05)	0.00%	0.457
**MEHP**	**Timing of specimen collection**				
	First trimester	1	1.05(0.72,1.53)		
	Second and third trimester	7	1.03(0.92,1.16)	33.40%	0.173
	**Study area**				

	Asia	4	1.18(0.94,1.47)	0.00%	0.675
	Europe	2	1.10(0.87,1.39)	0.00%	0.767
	North America	2	0.97(0.83,1.13)	65.20%	0.09
	**Children age**				
	<3	1	1.05(0.72,1.53)		
	≥3	7	1.03(0.92,1.16)	33.40%	0.173
**MBzP**	**Timing of specimen collection**				
	First trimester	1	1.11(0.76,1.63)		
	Second and third trimester	7	1.04(0.94,1.15)	41.90%	0.112
	**Study area**				
	Asia	1	1.07(0.91,1.26)		
	Europe	3	1.09(0.90,1.32)	0.00%	0.441
	North America	4	1.02(0.88,1.19)	60.00%	0.058
	**Children age**				
	≥3	7	1.04(0.94,1.15)	41.90%	0.112
	<3	1	1.11(0.76,1.63)		
**MnBP**	**Timing of specimen collection**				
	First trimester	1	1.10(0.76,1.60)		
	Second and third trimester	4	1.08(0.91,1.29)	0.00%	0.692
	**Study area**				
	Asia	1	1.22(0.88,1.69)		
	Europe	2	1.11(0.88,1.41)	0.00%	0.941
	North America	2	0.95(0.71,1.28)	0.00%	0.703

	**Children age**				
	≥3	4	1.08(0.91,1.29)	0.00%	0.692
	<3	1	1.10(0.76,1.60)		
**MiBP**	**Study area**				
	Asia	1	1.06(0.84,1.33)		
	Europe	1	0.96(0.71,1.30)		
	North America	3	0.93(0.82,1.06)	0.00%	0.99
	**Study area**				
**MCOP**	Europe	3	0.97(0.80,1.17)	49.70%	0.137
	North America	2	1.003(0.92,1.10)	0.00%	0.577
**MCPP**	**Study area**				
	Europe	1	1.15(0.85,1.56)		
	North America	2	1.044(0.93,1.17)	0.00%	0.765
**MEHHP**	**Timing of specimen collection**				
	First trimester	1	1.17(0.81,1.70)		
	Second and third trimester	4	1.102(0.91,1.33)	72.20%	0.013
	**Study area**				
	Asia	1	1.33(0.94,1.88)		
	Europe	2	1.12(0.89,1.42)	0.00%	0.773
	North America	2	1.055(0.81,1.38)	87.30%	0.005
	**Children age**				
	≥3	4	1.102(0.91,1.33)	72.20%	0.013
	<3	1	1.17(0.81,1.70)		

**MEOHP**	**Timing of specimen collection**				
	First trimester	1	1.18(0.81,1.72)		
	Second and third trimester	4	1.107(0.87,1.41)		
	**Study area**				
	Asia	1	1.32(0.93,1.87)		
	Europe	2	1.14(0.90,1.45)	0.00%	0.805
	North America	2	1.05(0.72,1.52)	92.00%	0
	**Children age**				
	≥3	4	1.11(0.87,1.41)	80.40%	0.002
	<3	1	1.18(0.81,1.72)		
**MECPP**	**Timing of specimen collection**				
	First trimester	1	1.07(0.74,1.55)		
	Second and third trimester	4	1.14(0.92,1.42)	75.50%	0.007
	**Study area**				
	Asia	1	1.41(1.02,2.00)		
	Europe	2	1.13(0.88,1.44)	0.00%	0.721
	North America	2	1.06(0.78,1.45)	88.00%	0.004
	**Children age**				
	≥3	4	1.14(0.92,1.42)	75.50%	0.007
	<3	1	1.07(0.74,1.55)		
**DEHP**	**Study area**				
	Europe	3	1.01(0.84,1.23)	33.40%	0.223
	North America	4	1.03(0.82,1.29)	70.00%	0.019

Supplementary Table S4. Subgroup analysis results on postnatal PAEs exposure and risk of wheeze.

Class of PAEs	Subgroup	No.	Subgroup analysis	Heterogeneity	
			OR (95% CI)	I^2 (%)	*p*-Value
**MEP**	**Study area**				
	Asia	8	1.04(0.91,1.19)	25.70%	0.224
	North America	2	1.1(0.88,1.37)	0.00%	0.781
	**Children age**				
	≥3	9	1.07(0.97,1.18)	0.00%	0.657
	<3	1	0.71(0.47,1.07)		
	**Study design**				
	Cross-sectional study	7	0.98(0.84,1.15)	15.70%	0.31
	Cohort study	3	1.13(0.99,1.30)	0.00%	0.93
**MBP**	**Children age**				
	≥3	4	0.86(0.58,1.28)	24.90%	0.262
	<3	1	1.17(0.58,2.33)		
	**Study design**				
	Cross-sectional study	3	1.1(0.64,1.90)	18.20%	0.295
	Cohort study	2	0.84(0.64,1.1)	0.00%	0.33
**MEHP**	**Study area**				
	Asia	6	0.99(0.81,1.21)	0.00%	0.554
	North America	1	1.46(1.14,1.87)		
	**Children age**				
	≥3	6	1.17(0.89,1.53)	43.70%	0.114

	<3	1	0.98(0.64,1.50)		
	**Study design**				
	Cross-sectional study	4	1.06(0.78,1.44)	0.00%	0.705
	Cohort study	3	1.18(0.79,1.78)	74.30%	0.02
**MBzP**	**Study area**				
	Asia	8	1.05(0.95,1.17)	0.00%	0.769
	North America	2	1.3(1.02,1.67)	0.00%	0.456
	**Children age**				
	≥3	9	1.09(0.99,1.20)	0.00%	0.53
	<3	1	1.08(0.76,1.53)		
	**Study design**				
	Cross-sectional study	7	1.08(0.96,1.21)	0.00%	0.725
	Cohort study	3	1.13(0.90,1.43)	40.40%	0.187
**MnBP**	**Study area**				
	Asia	5	1.06(0.83,1.35)	34.50%	0.191
	North America	2	0.76(0.31,1.82)	76.70%	0.038
	**Study design**				
	Cross-sectional study	5	0.96(0.71,1.30)	53.30%	0.073
	Cohort study	2	1.16(0.87,1.54)	0.00%	0.493
**MiBP**	**Study area**				
	Asia	3	1.00(0.69,1.44)	60.90%	0.077
	North America	2	1.11(0.85,1.45)	0.00%	0.932
	**Study design**				

	Cross-sectional study	3	0.92(0.63,1.35)	32.50%	0.227
	Cohort study	2	1.13(0.91,1.41)	0.00%	0.877
**MEHHP**	**Study area**				
	Asia	4	1.15(0.97,1.35)	0.00%	0.907
	North America	1	1.45(1.06,1.96)		
	**Study design**				
	Cross-sectional study	3	1.14(0.97,1.35)	0.00%	0.759
	Cohort study	2	1.39(1.05,1.82)	0.00%	0.542
**MEOHP**	**Study area**				
	Asia	4	1.29(0.79,2.12)	38.80%	0.179
	North America	2	1.47(1.17,1.85)	0.00%	0.797
	**Study design**				
	Cross-sectional study	2	1.56(0.94,2.61)	0.00%	0.942
	Cohort study	4	1.36(0.99,1.86)	40.60%	0.168
**DEHP**	**Study area**				
	Asia	4	1.00(0.70,1.42)	0.00%	0.505
	North America	2	1.02(0.41,2.52)	73.80%	0.051
	**Study design**				
	Cross-sectional study	4	0.95(0.65,1.40)	12.90%	0.328
	Cohort study	2	1.34(0.82,2.17)	19.00%	0.266

Supplementary Table S5. Subgroup analysis results on postnatal PAEs exposure from indoor dust and risk of wheeze.

**Class of PAEs**	**Subgroup**	**No.**	**Subgroup analysis**	**Heterogeneity**

			OR (95% CI)	I^2 (%)	*p*-Value
**MEP**	**Study area**				
	Asia	2	1.13(0.72,1.79)	42.00%	0.189
	North America	1	0.43(0.2,0.93)		
**MnBP**	**Study area**				
	Asia	2	0.97(0.85,1.1)	0.00%	0.836
	North America	1	1.35(0.61,2.99)		
	**Study design**				
	Cross-sectional study	1	1(0.7,1.43)		
	Case control	2	0.97(0.85,1.11)	0.00%	0.408
**MiBP**	**Study area**				
	Asia	3	1.15(0.98,1.35)	0.00%	0.51
	North America	1	1.33(0.61,2.89)		
	**Study design**				
	Cross-sectional study	1	1.15(0.81,1.63)		
	Case control	3	1.16(0.97,1.38)	0.00%	0.479
**MBzP**	**Study design**				
	Cross-sectional study	1	1.06(0.86,1.31)		
	Case control	2	0.94(0.8,1.12)	0.00%	0.771
**DEHP**	**Study area**				
	Asia	3	1.05(0.9,1.21)	0.00%	0.522
	Europe	1	3.7(1.39,9.84)		
	North America	1	2.87(1.19,6.93)		

	**Study design**				
	Cross-sectional study	1	1.18(0.79,1.76)		
	Case control	4	1.77(0.94,3.34)	75.10%	0.007

Supplementary Table S6. Subgroup analysis results on prenatal PAEs exposure and risk of asthma.

**Class of PAEs**	**Subgroup**	**No.**	**Subgroup**	**Heterogeneity**	
			**OR (95% CI)**	**I^2 (%)**	***p*-Value**
**MEP**	**Timing of specimen collection**				
	First trimester	6	0.98(0.89,1.07)	0.00%	0.712
	Second and third trimester	6	1.04(0.97,1.11)	0.00%	0.454
	Entire pregnancy	3	1.11(0.87,1.41)	0.00%	0.447
	**Study area**				
	Asia	6	0.98(0.89,1.07)	0.00%	0.712
	Europe	4	1.15(0.95,1.39)	0.00%	0.615
	North America	3	1.04(0.95,1.13)	1.70%	0.362
	Oceania	2	1(0.82,1.21)	31.30%	0.227
**MBP**	**Timing of specimen collection**				
	First trimester	4	0.98(0.71,1.36)	62.30%	0.047
	Second and third trimester	3	1.05(0.84,1.31)	55.30%	0.107
	Entire pregnancy	1	0.91(0.66,1.26)		
	**Study area**				
	Asia	4	0.98(0.71,1.36)	62.30%	0.047
	Europe	1	0.91(0.66,1.26)		

	North America	1	1.05(0.89,1.23)		
	Oceania	2	1.03(0.64,1.66)	77.60%	0.034
**MMP**	**Timing of specimen collection**				
	First trimester	5	1.04(0.82,1.33)	44.60%	0.124
	Entire pregnancy	1	1.04(0.8,1.35)		
	**Study area**				
	Asia	5	1.04(0.82,1.33)	44.60%	0.124
	Europe	1	1.04(0.8,1.35)		
**MBzP**	**Timing of specimen collection**				
	First trimester	6	1.16(0.96,1.41)	62.10%	0.022
	Second and third trimester	5	1.07(0.88,1.3)	45.80%	0.117
	Entire pregnancy	3	1.16(0.79,1.7)	61.40%	0.075
	**Study area**				
	Asia	6	1.16(0.96,1.41)	62.10%	0.022
	Europe	3	1.19(0.83,1.71)	50.00%	0.135
	North America	3	0.94(0.85,1.05)	0.00%	0.913
	Oceania	2	1.37(1.03,1.83)	11.00%	0.289
**MnBP**	**Timing of specimen collection**				
	First trimester	1	1.78(1.18,2.69)		
	Second and third trimester	3	1.1(0.83,1.46)	20.80%	0.283
	Entire pregnancy	3	1.03(0.79,1.34)	26.30%	0.257
	**Study area**				
	Asia	1	1.78(1.18,2.69)		

	Europe	3	1.08(0.82,1.42)	11.20%	0.324
	North America	2	0.89(0.66,1.2)	0.00%	0.676
	Oceania	1	1.31(0.96,1.78)		
**MiBP**	**Timing of specimen collection**				
	Second and third trimester	4	1(0.87,1.14)	0.00%	0.422
	Entire pregnancy	3	0.91(0.73,1.13)	5.40%	0.347
	**Study area**				
	Europe	3	0.78(0.6,1.02)	0.00%	0.616
	North America	3	1(0.88,1.15)	0.00%	0.712
	Oceania	1	1.17(0.79,1.74)		
**MEHP**	**Timing of specimen collection**				
	First trimester	5	0.91(0.68,1.22)	60.80%	0.037
	Second and third trimester	3	1.03(0.78,1.36)	65.10%	0.057
	Entire pregnancy	2	0.86(0.48,1.55)	73.50%	0.052
	**Study area**				
	Asia	5	0.91(0.68,1.22)	60.80%	0.037
	Europe	2	0.86(0.46,1.55)	73.50%	0.052
	North America	1	0.88(0.80,0.97)		
	Oceania	2	1.23(0.95,1.58)	0.00%	0.965
**MCNP**	**Timing of specimen collection**				
	Second and third trimester	1	0.91(0.81,1.03)		
	Entire pregnancy	2	1.34(0.94,1.92)	0.00%	0.606
	**Study area**				

	Europe	1	1.46(0.91,2.35)		
	North America	2	0.93(0.81,1.07)	4.40%	0.306
**MCOP**	**Timing of specimen collection**				
	Second and third trimester	4	0.97(0.87,1.09)	8.70%	0.35
	Entire pregnancy	3	1.18(0.88,1.59)	50.40%	0.133
	**Study area**				
	Europe	3	0.93(0.73,1.18)	0.00%	0.475
	North America	2	1.2(0.77,1.86)	85.60%	0.008
	Oceania	2	1.05(0.78,1.41)	34.70%	0.216
**MCPP**	**Timing of specimen collection**				
	Second and third trimester	3	1.02(0.9,1.15)	0.00%	0.985
	Entire pregnancy	3	1.07(0.86,1.35)	0.00%	0.564
	**Study area**				
	Europe	2	1.05(0.8,1.37)	0.60%	0.316
	North America	3	1.03(0.92,1.15)	0.00%	0.848
	Oceania	1	1.02(0.32,3.25)		
**MEHHP**	**Timing of specimen collection**				
	First trimester	6	1.06(0.86,1.31)	17.30%	0.302
	Second and third trimester	2	1.07(0.81,1.42)	77.30%	0.036
	Entire pregnancy	2	0.98(0.75,1.27)	0.00%	0.888
	**Study area**				
	Asia	6	1.06(0.86,1.31)	17.30%	0.302
	Europe	2	0.98(0.75,1.27)	0.00%	0.888

	North America	1	0.95(0.86,1.05)		
	Oceania	1	1.27(0.99,1.64)		
**MEOHP**	**Timing of specimen collection**				
	First trimester	5	1.12(0.73,1.73)	57.20%	0.053
	Second and third trimester	2	1.11(0.73,1.7)	87.80%	0.004
	Entire pregnancy	2	1.03(0.8,1.34)	0.00%	0.55
	**Study area**				
	Asia	5	1.12(0.73,1.73)	57.20%	0.053
	Europe	2	1.03(0.8,1.34)	0.00%	0.55
	North America	1	0.91(0.79,1.05)		
	Oceania	1	1.4(1.08,1.81)		
**MECPP**	**Timing of specimen collection**				
	Second and third trimester	3	1.16(0.84,1.59)	69.60%	0.037
	Entire pregnancy	2	1.07(0.81,1.41)	0.00%	0.782
	**Study area**				
	Europe	2	1.07(0.81,1.41)	0.00%	0.782
	North America	1	0.96(0.84,1.1)		
	Oceania	2	1.4(1.08,1.82)	0.00%	0.805
**DEHP**	**Timing of specimen collection**				
	Second and third trimester	6	1.02(0.86,1.21)	40.20%	0.137
	Entire pregnancy	3	0.84(0.68,1.04)	0.00%	0.78
	**Study area**				
	Europe	3	0.89(0.7,1.11)	0.00%	0.59

	North America	3	0.89(0.79,1.02)	0.00%	0.836
	Oceania	3	1.14(0.84,1.55)	53.20%	0.118

Supplementary Table S7. Subgroup analysis results on postnatal PAEs exposure and risk of asthma.

**Class of PAEs**	**Subgroup**	**No.**	**Subgroup analysis**	**Heterogeneity**	
			**OR (95% CI)**	**I^2 (%)**	***p*-Value**
**MEP**	**Study area**				
	Asia	5	1.04(0.9,1.19)	0.00%	0.455
	Europe	4	0.92(0.63,1.35)	0.00%	0.754
	**Study design**				
	Cross-sectional study	4	0.92(0.63,1.35)	0.00%	0.754
	Case control	5	1.04(0.9,1.19)	0.00%	0.455
**MBP**	**Study area**				
	Asia	2	1.03(0.86,1.24)	0.00%	0.519
	Europe	1	0.51(0.11,2.35)		
	**Study design**				
	Cross-sectional study	1	0.51(0.11,2.35)		
	Case control	2	1.03(0.86,1.24)	0.00%	0.519
**MBzP**	**Study area**				
	Asia	5	1.07(0.88,1.31)	0.00%	0.842
	Europe	4	1.06(0.79,1.41)	31.20%	0.214
	**Study design**				
	Cross-sectional study	4	1.06(0.79,1.41)	0.00%	0.842

	Case control	5	1.07(0.88,1.31)	31.20%	0.214
**MiBP**	**Study area**				
	Asia	3	1.13(0.87,1.46)	0.00%	0.91
	Europe	4	1.08(0.76,1.52)	16.80%	0.307
	**Study design**				
	Cross-sectional study	4	1.08(0.76,1.52)	16.80%	0.307
	Case control	3	1.13(0.87,1.46)	0.00%	0.91
**MnBP**	**Study area**				
	Asia	4	1.31(0.88,1.95)	30.20%	0.231
	Europe	4	0.89(0.47,1.72)	63.00%	0.044
	**Study design**				
	Cross-sectional study	4	0.89(0.47,1.72)	63.00%	0.044
	Case control	4	1.31(0.88,1.95)	30.20%	0.231
**MEHP**	**Study area**				
	Asia	3	1.68(1.11,2.56)	0.00%	0.691
	Europe	2	0.54(0.28,1.06)	0.00%	0.549
	**Study design**				
	Cross-sectional study	2	0.54(0.28,1.06)	0.00%	0.549
	Case control	3	1.68(1.11,2.56)	0.00%	0.691
**MCPP**	**Study area**				
	Asia	3	0.79(0.51,1.22)	0.00%	0.824
	Europe	1	1.20(0.66,2.19)		
	**Study design**				

	Cross-sectional study	1	1.20(0.66,2.19)		
	Case control	3	0.79(0.51,1.22)	17.70%	0.824
**MCNP**	**Study area**				
	Asia	3	0.67(0.35,1.34)	64.60%	0.059
	Europe	1	2.20(1.21,4.02)		
	**Study design**				
	Cross-sectional study	3	0.67(0.35,1.34)	64.60%	0.059
	Case control	1	2.20(1.21,4.02)		
**MCOP**	**Study area**				
	Asia	2	1.02(0.66,1.58)	17.10%	0.272
	Europe	1	1.90(1.05,3.45)		
	**Study design**				
	Cross-sectional study	1	1.9(1.05,3.45)		
	Case control	2	1.02(0.66,1.58)	17.10%	0.272
**MEHHP**	**Study area**				
	Asia	3	1.32(1.01,1.73)	37.80%	0.201
	Europe	1	0.84(0.37,1.92)		
	**Study design**				
	Cross-sectional study	1	0.84(0.37,1.92)		
	Case control	3	1.32(1.01,1.73)	37.80%	0.201
**MEOHP**	**Study area**				
	Asia	2	1.46(0.81, 2.64)	54.40%	0.139
	Europe	2	0.55(0.27,1.14)	0.00%	0.982

	**Study design**				
	Cross-sectional study	2	0.55(0.27,1.14)	0.00%	0.982
	Case control	2	1.46(0.81, 2.64)	54.40%	0.139
**MECPP**	**Study area**				
	Asia	2	0.86(0.57,1.31)	0.00%	0.919
	Europe	2	0.56(0.27,1.166)	0.00%	0.84
	**Study design**				
	Cross-sectional study	2	0.56(0.27,1.166)	0.00%	0.84
	Case control	2	0.86(0.57,1.31)	0.00%	0.919
**DEHP**	**Study area**				
	Asia	4	0.85(0.38,1.91)	84.20%	0
	Europe	2	0.49(0.03,8.17)	93.30%	0
	**Study design**				
	Cross-sectional study	2	0.49(0.03,8.17)	93.30%	0
	Case control	4	0.85(0.38,1.91)	84.20%	0

Supplementary Table S8. Subgroup analysis results on postnatal PAEs exposure from indoor dust and risk of asthma.

**Class of PAEs**	**Subgroup**	**No.**	**Subgroup analysis**	**Heterogeneity**	
			**OR (95% CI)**	**I^2 (%)**	***p*-Value**
**MEP**	**Study design**				
	Cross-sectional study	1	1.25(0.41,3.82)		
	Case control	2	1.23(0.96,1.58)	0.00%	0.694
**MBzP**	**Study design**				

	Cross-sectional study	2	2(1.03,3.86)	0.00%	0.631
	Case control	2	0.95(0.8,1.12)	0.00%	0.752
**MiBP**	**Study design**				
	Cross-sectional study	1	8.94(0.86,92.96)		
	Case control	2	1.26(1.05,1.52)	0.10%	0.317
**DEHP**	**Study design**				
	Cross-sectional study	3	2.32(1.25,4.3)	0.00%	0.964
	Case control	2	1.25(0.84,1.87)	44.90%	0.178

Supplementary Table S9. Subgroup analysis results on prenatal PAEs exposure and risk of eczema.

**Class of PAEs**	**Subgroup**	**No.**	**Subgroup analysis**	**Heterogeneity**	
			**OR (95% CI)**	**I^2 (%)**	***p*-Value**
**MEP**	**Study area**				
	Asia	3	1.05(0.95,1.16)	0.00%	0.886
	North America	4	0.92(0.78,1.08)	8.70%	0.35
	**Children age**				
	≥3	6	0.99(0.9,1.1)	12.10%	0.338
	<3	1	1.09(0.27,4.39)		
**MBzP**	**Timing of specimen collection**				
	First trimester	2	1.06(0.93,1.21)	0.00%	0.786
	Second and third trimester	6	1.24(0.99,1.55)	59.80%	0.029
	Entire pregnancy	1	1.34(0.86,2.09)		
	**Study area**				

	Asia	3	1.22(0.84,1.78)	52.20%	0.123
	North America	6	1.19(1.02,1.39)	38.60%	0.148
	**Children age**				
	≥3	7	1.06(0.98,1.15)	0.00%	0.774
	<3	2	1.61(1.08,2.39)	9.50%	0.293
**MiBP**	**Timing of specimen collection**				
	First trimester	1	1.02(0.56,1.87)		
	Second and third trimester	5	1.11(0.88,1.4)	44.90%	0.123
	**Study area**				
	Asia	2	1.15(1,1.31)	0.00%	0.361
	North America	4	1.14(0.79,1.63)	53.10%	0.094
	**Children age**				
	≥3	5	1.13(0.91,1.4)	39.50%	0.158
	<3	1	0.84(0.43,1.66)		
**MnBP**	**Timing of specimen collection**				
	First trimester	1	1.09(0.79,1.5)		
	Second and third trimester	5	0.99(0.79,1.24)	0.00%	0.432
	**Study area**				
	Asia	2	0.86(0.55,1.33)	0.00%	0.589
	North America	4	1.06(0.87,1.31)	0.20%	0.391
	**Children age**				
	≥3	5	1.04(0.86,1.25)	0.00%	0.503
	<3	1	0.67(0.25,1.82)		

**MEHP**	**Timing of specimen collection**				
	First trimester	2	1.03(0.8,1.32)	53.10%	0.144
	Second and third trimester	7	0.91(0.76,1.08)	22.10%	0.261
	**Study area**				
	Asia	6	0.89(0.76,1.05)	14.20%	0.323
	North America	3	1.07(0.84,1.37)	42.70%	0.175
	**Children age**				
	≥3	7	0.97(0.86,1.1)	26.40%	0.227
	<3	2	0.69(0.42,1.14)	0.00%	0.528
**MCOP**	**Timing of specimen collection**				
	Second and third trimester	2	1.09(0.98,1.23)		
	Entire pregnancy	2	1.06(0.7,1.6)		
	**Study area**				
	Asia	2	1(0.83,1.21)	60.00%	0.114
	North America	2	1.14(0.95,1.38)	0.00%	0.366
**MCPP**	**Timing of specimen collection**				
	Second and third trimester	2	1.05(0.92,1.21)	0.00%	0.893
	Entire pregnancy	1	0.83(0.57,1.21)		
	**Study area**				
	Asia	1	1.05(0.91,1.22)		
	North America	2	0.95(0.72,1.24)	0.00%	0.336
**MEHHP**	**Timing of specimen collection**				
	First trimester	2	1.08(0.91,1.27)	10.50%	0.291

	Second and third trimester	1	0.99(0.87,1.13)		
	Entire pregnancy	1	1.53(1.15,2.03)		
	**Study area**				
	Asia	2	1.21(0.79,1.85)	86.60%	0.006
	North America	2	1.08(0.91,1.27)	10.50%	0.291
	**Children age**				
	≥3	3	1.02(0.93,1.13)	0.00%	0.425
	<3	1	1.53(1.15,2.03)		
**MEOHP**	**Timing of specimen collection**				
	First trimester	2	1.11(0.86,1.44)	49.00%	0.161
	Second and third trimester	1	1.01(0.88,1.16)		
	Entire pregnancy	1	1.49(1.11,2.01)		
	**Study area**				
	Asia	2	1.2(0.82,1.75)	81.50%	0.02
	North America	2	1.11(0.86,1.44)	49.00%	0.161
	**Children age**				
	≥3	3	1.04(0.93,1.17)	11.80%	0.322
	<3	1	1.49(1.11,2.01)		
**MECPP**	**Timing of specimen collection**				
	First trimester	1	0.98(0.85,1.14)		
	Second and third trimester	2	1.1(0.88,1.38)	43.50%	0.183
	**Study area**				
	Asia	1	0.98(0.85,1.14)		

	North America	2	1.1(0.88,1.38)	43.50%	0.183
**DEHP**	**Timing of specimen collection**				
	First trimester	1	1(0.83,1.2)		
	Second and third trimester	3	0.98(0.94,1.03)	0.00%	0.611
	Entire pregnancy	2	1.03(0.87,1.24)	0.00%	0.38
	**Study area**				
	Asia	2	0.98(0.94,1.03)	0.00%	0.841
	North America	4	1.03(0.9,1.18)	0.00%	0.648

Supplementary Table S10. Subgroup analysis results on postnatal PAEs exposure and risk of eczema.

**Class of PAEs**	**Subgroup**	**No.**	**Subgroup analysis**	**Heterogeneity**	
			**OR (95% CI)**	**I^2 (%)**	***p*-Value**
**MEP**	**Study area**				
	Asia	7	0.94(0.83,1.07)	0.00%	0.627
	Europe	1	2.27(1.12,4.61)		
	**Children age**				
	≥3	6	1.07(0.85,1.35)	45.80%	0.1
	<3	2	0.91(0.65,1.27)	0.00%	0.396
	**Study design**				
	Cross-sectional study	2	2.07(1.17,3.68)	0.00%	0.672
	Cohort study	6	0.93(0.82,1.06)	0.00%	0.734
**MBP**	**Children age**				
	≥3	4	0.91(0.73,1.13)	0.00%	0.886

	<3	2	0.96(0.58,1.58)	0.00%	0.555
**MBzP**	**Study area**				
	Asia	12	1.09(0.98,1.22)	28.20%	0.169
	Europe	1	1.43(0.72,2.86)		
	**Children age**				
	≥3	11	1.1(1.02,1.2)	0.00%	0.463
	<3	2	1.36(0.48,3.83)	81.80%	0.019
	**Study design**				
	Cross-sectional study	7	1.13(1.03,1.24)	0.00%	0.465
	Cohort study	6	1.08(0.85,1.37)	41.70%	0.127
**MnBP**	**Study area**				
	Asia	8	1.15(0.97,1.35)	24.90%	0.23
	Europe	1	0.62(0.31,1.24)		
	**Study design**				
	Cross-sectional study	8	1.14(0.94,1.4)	40.50%	0.109
	Cohort study	1	0.98(0.63,1.52)		
**MiBP**	**Study area**				
	Asia	3	1.03(0.75,1.42)	66.80%	0.049
	Europe	1	0.97(0.48,1.95)		
	**Study design**				
	Cross-sectional study	3	1.12(0.63,1.97)	60.80%	0.078
	Cohort study	1	1.04(0.84,1.29)		
**MEHP**	**Study area**				

	Asia	7	1.03(0.89,1.18)	0.00%	0.523
	Europe	1	0.93(0.46,1.88)		
	**Children age**				
	≥3	6	1.02(0.88,1.19)	0.00%	0.421
	<3	2	1.03(0.71,1.49)	0.00%	0.599
	**Study design**				
	Cross-sectional study	2	1.44(0.54,3.84)	61.50%	0.107
	Cohort study	6	1.01(0.87,1.16)	0.00%	0.856
**MEHHP**	**Study area**				
	Asia	7	1.25(1.06,1.47)	0.418	
	Europe	1	0.68(0.34,1.36)		
	**Study design**				
	Cross-sectional study	7	1.27(1.04,1.55)	13.80%	0.325
	Cohort study	1	0.93(0.62,1.4)		
**MEOHP**	**Study area**				
	Asia	7	1.34(1.11,1.61)	21.50%	0.265
	Europe	1	0.79(0.39,1.6)		
	**Study design**				
	Cross-sectional study	7	1.37(1.19,1.59)	0.00%	0.46
	Cohort study	1	0.85(0.55,1.33)		
**MECPP**	**Study area**				
	Asia	5	1.2(0.99,1.46)		
	Europe	1	0.78(0.39,1.56)		

	**Study design**				
	Cross-sectional study	5	1.21(0.99,1.48)	0.00%	0.559
	Cohort study	1	0.92(0.56,1.52)		
**DEHP**	**Study design**				
	Cross-sectional study	5	1.32(1.1,1.57)	0.00%	0.532
	Cohort study	1	2.92(0.82,10.38)		

Supplementary Table S11. Subgroup analysis results on postnatal PAEs exposure from indoor dust and risk of eczema.

**Class of PAEs**	**Subgroup**	**No.**	**Subgroup analysis**	**Heterogeneity**	
			**OR (95% CI)**	**I^2 (%)**	***p*-Value**
MBzP	**Study area**				
	Asia	4	1.08(0.8,1.47)	74.00%	0.009
	Europe	1	2.56(1.24,5.3)		
	**Study design**				
	Cross-sectional study	3	2.17(0.66,7.22)	74.20%	0.021
	Case control	2	1.42(0.51,3.96)	87.30%	0.005
MiBP	**Study design**				
	Cross-sectional study	2	0.15(0.22,44.45)	84.80%	0.01
	Case control	1	0.95(0.85,1.07)		
MnBP	**Study design**				
	Cross-sectional study	2	1.05(0.78,1.41)	0.00%	0.776
	Case control	1	0.94(0.86,1.02)		
MCOP	**Study design**				

	Cross-sectional study	2	1.18(0.92,1.52)	0.00%	0.784
	Case control	1	0.98(0.86,1.12)		
DEHP	**Study area**				
	Asia	4	0.94(0.86,1.04)	0.00%	0.765
	Europe	2	1.72(0.96,3.1)	0.00%	0.427
	**Study design**				
	Cross-sectional study	3	0.97(0.72,1.31)	0.00%	0.573
	Case control	3	1.25(0.74,2.08)	56.30%	0.101

Supplementary Table S12. Subgroup analysis results on prenatal PAEs exposure and risk of rhinitis.

**Class of PAEs**	**Subgroup**	**No.**	**Subgroup analysis**	**Heterogeneity**	
			**OR (95% CI)**	**I^2 (%)**	**p-Value**
**MEP**	**Timing of specimen collection**				
	First trimester	1	1.05(0.91,1.21)		
	Second and third trimester	2	0.81(0.56,1.18)	45.80%	0.174
	**Study area**				
	Asia	1	1.05(0.91,1.21)		
	Europe	2	0.81(0.56,1.18)	45.80%	0.174
**MBzP**	**Timing of specimen collection**				
	First trimester	2	1.09(0.99,1.19)	0.00%	0.949
	Second and third trimester	1	0.88(0.53,1.47)		
	**Study area**				
	Asia	2	1.09(0.99,1.19)	0.00%	0.949

	Europe	1	0.88(0.53,1.47)		
**MEHP**	**Timing of specimen collection**				
	First trimester	2	1.12(0.97,1.3)	0.00%	0.474
	Second and third trimester	1	1.47(0.97,2.23)		
	**Study area**				
	Asia	2	1.12(0.97,1.3)	0.00%	0.474
	Europe	1	1.47(0.97,2.23)		

Supplementary Table S13. Subgroup analysis results on postnatal PAEs exposure and risk of rhinitis.

**Class of PAEs**	**Subgroup**	**No.**	**Subgroup analysis**	**Heterogeneity**	
			**OR (95% CI)**	**I^2 (%)**	***p*-Value**
**MEP**	**Study area**				
	Asia	5	0.92(0.8,1.06)	0.00%	0.622
	Europe	1	1.6(0.75,3.41)		
	North America	1	0.89(0.65,1.22)		
	**Children age**				
	≥3	6	0.95(0.82,1.09)	0.00%	0.512
	<3	1	0.85(0.63,1.15)		
	**Study design**				
	Cross-sectional study	3	0.98(0.75,1.29)	0.20%	0.367
	Cohort study	4	0.92(0.79,1.06)	0.00%	0.478
**MBP**	**Children age**				
	≥3	3	1(0.69,1.46)	47.80%	0.147

	<3	1	1.84(1.06,3.19)		
**MBzP**	**Study area**				
	Asia	9	1.03(0.98,1.08)	0.00%	0.495
	Europe	1	1.18(0.56,2.48)		
	North America	1	1.02(0.62,1.67)		
	**Children age**				
	≥3	10	1.02(0.97,1.08)	0.00%	0.608
	<3	1	1.09(0.85,1.39)		
	**Study design**				
	Cross-sectional study	7	1.02(0.95,1.1)	16.10%	0.307
	Cohort study	4	1.04(0.92,1.16)		
**MnBP**	**Study area**				
	Asia	6	1.05(0.94,1.17)	0.00%	0.561
	Europe	1	1.36(0.64,2.89)		
	North America	1	0.83(0.46,1.51)		
	**Study design**				
	Cross-sectional study	7	1.05(0.94,1.16)	0.00%	0.57
	Cohort study	1	1.18(0.67,2.08)		
**MiBP**	**Study area**				
	Asia	3	1.11(0.83,1.48)	57.00%	0.098
	Europe	1	1.07(0.52,2.21)		
	North America	1	0.84(0.53,1.33)		
	**Study design**				

	Cross-sectional study	4	1.07(0.78,1.46)	43.80%	0.148
	Cohort study	1	1.03(0.78,1.36)		
**MEHP**	**Study area**				
	Asia	5	0.96(0.82,1.12)	0.00%	0.8
	Europe	1	0.71(0.34,1.48)		
	**Children age**				
	≥3	5	0.93(0.78,1.11)	0.00%	0.727
	<3	1	1.01(0.75,1.37)		
	**Study design**				
	Cross-sectional study	2	0.96(0.54,1.71)	0.256	
	Cohort study	4	0.95(0.81,1.11)	0.806	
**MCOP**	**Study area**				
	Asia	3	1.12(0.98,1.29)	0.00%	0.445
	North America	1	1.4(0.83,2.37)		
**MCNP**	**Study area**				
	Asia	3	1.16(1.03,1.32)	0.339	
	North America	1	1.23(0.71,2.13)		
**MCPP**	**Study area**				
	Europe	3	0.97(0.83,1.14)	0.00%	0.625
	North America	1	1.02(0.65,1.59)		
**MEHHP**	**Study area**				
	Asia	5	1.21(1.07,1.36)	0.00%	0.824
	Europe	1	0.7(0.33,1.49)		

	**Study design**				
	Cross-sectional study	5	1.2(1.06,1.35)	0.00%	0.499
	Cohort study	1	1.1(0.66,1.83)		
**MEOHP**	**Study area**				
	Asia	5	1.17(1.05,1.3)	0.00%	0.86
	Europe	1	0.65(0.3,1.41)		
	**Study design**				
	Cross-sectional study	5	1.17(1.05,1.3)	0.00%	0.598
	Cohort study	1	0.91(0.52,1.6)		
**MECPP**	**Study area**				
	Asia	5	1.13(1,1.28)	0.00%	0.906
	Europe	1	0.89(0.41,1.92)		
	**Study design**				
	Cross-sectional study	5	1.13(1,1.28)	0.894	
	Cohort study	1	0.95(0.51,1.79)		
**DEHP**	**Study area**				
	Asia	6	1.19(1.04,1.34)	0.00%	0.841
	North America	1	1.52(0.86,2.67)		
	**Study design**				
	Cross-sectional study	6	1.2(1.06,1.36)	0.00%	0.74
	Cohort study	1	1.09(0.4,2.98)		

Supplementary Table S14. Subgroup analysis results on postnatal PAEs exposure from indoor dust and risk of rhinitis.

Class of PAEs	Subgroup	No.	Subgroup analysis	Heterogeneity	
			OR (95% CI)	I^2 (%)	*p*-Value
**MEP**	**Study design**				
	Cross-sectional study	1	1.68(0.67,4.22)		
	Case control	2	0.99(0.67,1.47)	29.60%	0.233
**MBzP**	**Study area**				
	Asia	4	1.55(0.85,2.81)	57.30%	0.071
	Europe	1	3.04(1.34,6.89)		
	**Study design**				
	Cross-sectional study	2	2.72(1.09,6.81)	0.00%	0.773
	Case control	3	1.63(0.78,3.41)	78.70%	0.009
**MiBP**	**Study design**				
	Cross-sectional study	1	2.3(0.6,8.85)		
	Case control	2	1.01(0.87,1.17)	0.00%	0.857
**DEHP**	**Study area**				
	Asia	4	1.72(0.85,3.46)	67.80%	0.026
	Europe	2	1.74(0.96,3.15)	0.00%	0.628
	**Study design**				
	Cross-sectional study	2	2.46(0.98,6.2)	0.00%	0.523
	Case control	4	1.51(0.89,2.57)	65.50%	0.034

Supplementary Table S15. The E-value of meta-analysis results.

	**wheeze**		**asthma**	**eczema**		**rhinitis**		
	prenatal PAEs exposure	postnatal PAEs exposure	exposure to PAEs from indoor dust	prenatal PAEs exposure	postnatal PAEs exposure	prenatal PAEs exposure	postnatal PAEs exposure	exposure to PAEs from indoor dust
**MCOP**					1.9		1.57	
**MEHHP**		1.71					1.67	
**MEOHP**		2.34			1.9			
**MECPP**							1.51	
**MEHP**						1.59		
**MBzP**				1.62				3.12
**DEHP**			2.39		1.99		1.69	2.71
**Overall**	16.96	19.49	6.07	20.44	8.48		8.9	6.24
